# Adropin effects on granulosa cell function and oocyte maturation in pigs^†^

**DOI:** 10.1093/biolre/ioag174

**Published:** 2026-08-10

**Authors:** Patrycja Kurowska, Alicja Łapa, Jakub Chatys, Konrad Król, Michalina Cielińska, Karolina Kubicka, Marek Skrzypski, Monika Trzcińska, Lechosław Gajda, Joelle Dupont, Agnieszka Rak

**Affiliations:** Laboratory of Physiology and Toxicology of Reproduction, Institute of Zoology and Biomedical Research, Faculty of Biology, Jagiellonian University in Krakow, Gronostajowa 9, 30-387 Krakow, Poland; Laboratory of Physiology and Toxicology of Reproduction, Institute of Zoology and Biomedical Research, Faculty of Biology, Jagiellonian University in Krakow, Gronostajowa 9, 30-387 Krakow, Poland; Laboratory of Physiology and Toxicology of Reproduction, Institute of Zoology and Biomedical Research, Faculty of Biology, Jagiellonian University in Krakow, Gronostajowa 9, 30-387 Krakow, Poland; Laboratory of Physiology and Toxicology of Reproduction, Institute of Zoology and Biomedical Research, Faculty of Biology, Jagiellonian University in Krakow, Gronostajowa 9, 30-387 Krakow, Poland; Laboratory of Physiology and Toxicology of Reproduction, Institute of Zoology and Biomedical Research, Faculty of Biology, Jagiellonian University in Krakow, Gronostajowa 9, 30-387 Krakow, Poland; Laboratory of Physiology and Toxicology of Reproduction, Institute of Zoology and Biomedical Research, Faculty of Biology, Jagiellonian University in Krakow, Gronostajowa 9, 30-387 Krakow, Poland; Department of Animal Physiology and Biochemistry, Poznan University of Life Sciences, Wołyńska 35, 60-637 Poznan, Poland; Department of Reproductive Biotechnology and Cryoconservation, National Research Institute of Animal Production, Krakowska 1, Balice, 32-083 Krakow, Poland; Department of Reproductive Biotechnology and Cryoconservation, National Research Institute of Animal Production, Krakowska 1, Balice, 32-083 Krakow, Poland; National Research Institute for Agriculture, Food and the Environment, Unité Physiologie de la Reproduction et des Comportements, 37-380 Nouzilly, France; Laboratory of Physiology and Toxicology of Reproduction, Institute of Zoology and Biomedical Research, Faculty of Biology, Jagiellonian University in Krakow, Gronostajowa 9, 30-387 Krakow, Poland

**Keywords:** adropin, ovary, oocyte maturation, proliferation, autophagy

## Abstract

Adropin, a peptide involved in energy homeostasis, acts *via* G protein-coupled receptor 19 (GPR19). This study examined the expression of adropin/GPR19 in porcine ovarian follicles and assessed the effects of adropin on granulosa cells (Gc) function and oocyte maturation in vitro. Levels of adropin/GPR19 were analyzed in porcine ovarian follicles and follicular fluid during the estrous cycle. Then, Gc isolated from medium-sized follicles were cultured with gonadotropins, steroids, or adropin (0.1–100 nM) to evaluate adropin/GPR19 expression, Gc function, and signaling pathways using pharmacological inhibitors. Additionally, the effect of adropin on oocyte maturation was assessed after in vitro maturation of cumulus-oocyte complexes from prepubertal pigs. We noted that adropin expression increased in the whole porcine ovarian follicle, while GPR19 decreased during the estrous cycle. Adropin protein expression and secretion to culture medium and GPR19 protein increased with gonadotropin and steroid treatment. Both proteins are localized to Gc and theca cells. Functionally, adropin reduced Gc viability *via* AKT signaling, inhibited proliferation and cell cycle progression *via* ERK1/2, suppressed estradiol secretion and CYP19A1 expression *via* PKA, increased progesterone secretion, cleaved caspase-3 expression and its activity, and inhibited autophagy-related markers. Adropin simultaneously enhancing oocyte maturation, progesterone secretion, and lipid utilization in cumulus-oocyte complexes. These findings indicate that adropin may regulate porcine follicular development through its actions on Gc and oocytes.

## Teaser

Adropin modulates porcine follicular development by suppressing granulosa cell growth, steroidogenesis, and autophagy, while promoting oocyte maturation.

## Graphical Abstract

**Graphical Abstract:**
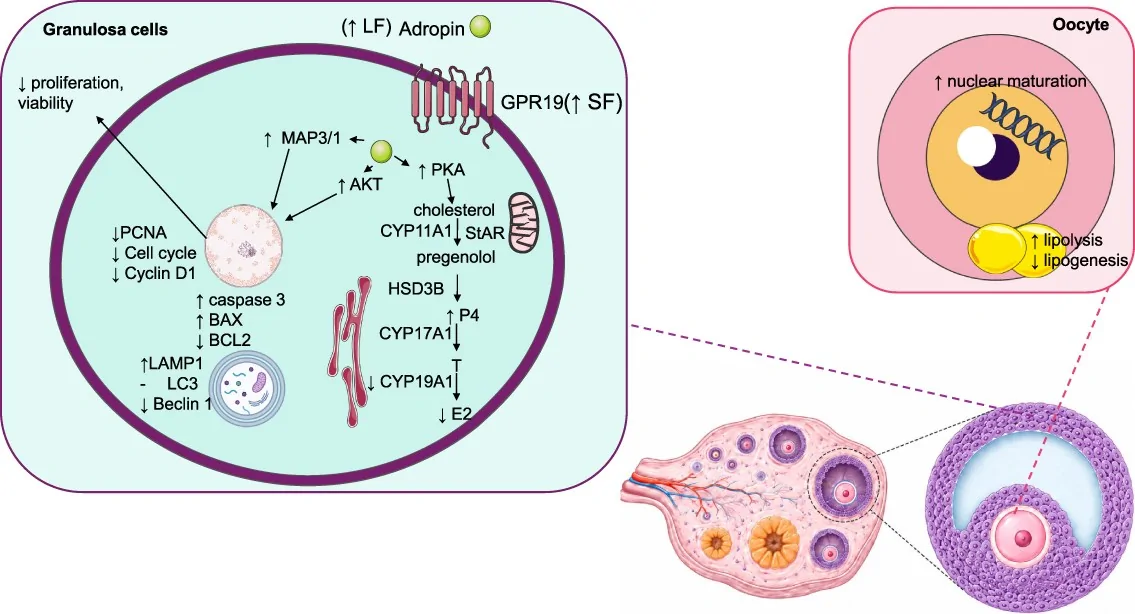

## Introduction

Adropin is a recently identified hormonally active secreted polypeptide, discovered by Kumar et al. [1] in the liver of mice. It is encoded by the Energy Homeostasis Associated (*ENHO*) gene, which spans 1948 base pairs and is involved in energy homeostasis. This gene is located on chromosome 9 in both humans and mice [1]. Adropin is a secreted protein composed of 43 amino acids, with the first 33 residues forming only the signal sequence. Its amino acid composition is identical among humans, mice, and rats [1, 2]. The presence of adropin has been detected in various tissues, including the heart, lungs, kidneys, and muscles [1]. The regulation of adropin levels is influenced by both metabolic and hormonal factors, e.g., a lower serum adropin concentration in humans has been linked to obesity, aging, and insulin resistance, whereas weight loss has been shown to elevate its levels [3], while in polycystic ovarian syndrome (PCOS) adropin level is decreased [4].

The biological effects of adropin are mediated through its interaction with GPR19, a seven-transmembrane domain, G-protein-coupled receptor [5]. In humans, the GPR19 gene is found on chromosome 12 [6], whereas in mice on chromosome 6 [7]. The receptor is expressed in multiple tissues, including the brain, liver, testes, ovaries, and embryonic structures in mice [7]. By binding to GPR19, adropin exerts pleiotropic effects, primarily in the regulation of energy metabolism, such as inhibiting water intake [8]. Furthermore, administration of adropin to obese mice improved glucose tolerance and decreased insulin resistance [9]. Additionally, adropin has been shown to suppress the expression of lipogenic genes in the liver of mice [1]. Interestingly, it promotes the proliferation of 3T3-L1 preadipocytes in mice [10] and murine endothelial cells [11]. Some findings also suggest that adropin plays a crucial role in endocrine regulation across different tissues, such as inhibiting glucose-stimulated insulin secretion in insulinoma INS-1E cells and isolated pancreatic islets from rats [12].

Moreover, studies have linked adropin to the regulation of female reproductive functions. In the mouse ovary, the expression of both adropin and GPR19 varies with the estrous cycle, reaching its peak during the estrous phase. Adropin has been found to stimulate progesterone (P4) synthesis and neovascularization, which ultimately facilitates corpus luteum formation [13]. It also plays a role in early folliculogenesis by enhancing granulosa cells (Gc) proliferation and estradiol (E2) synthesis in mice [14]. However, despite evidence indicating the involvement of adropin in ovarian function in rodents, its role in porcine follicular function and oocyte maturation remains unknown. Identification of metabolic factors regulating Gc function and oocyte competence may improve our understanding of the relationship between metabolic status and female fertility, as well as support the development of assisted reproductive technologies and in vitro maturation systems. Moreover, due to similarities between porcine and human ovarian physiology, the pig represents a valuable translational model for reproductive studies.

Thus, we hypothesize that adropin and GPR19 are expressed in porcine ovarian follicles and that adropin directly regulates porcine Gc function and oocyte maturation. Hence, in the present study, we investigated: (i) expression of adropin and GPR19 in ovarian follicle during estrous cycle, adropin concentration in follicular fluid (FF), its regulation by gonadotropins and steroids as well as its immunolocalization in the porcine ovary, (ii) the in vitro effect of adropin on Gc proliferation and viability, cell cycle progression, apoptosis, autophagy, steroidogenesis, and oocytes in vitro maturation, (iii) involvement of mitogen activated kinase (MAP3/1), protein kinase B (AKT) and A (PKA) in adropin action in porcine Gc.

## Material and methods

### Sample collection

Ovaries were collected from 7–8-month-old cross-breed sexually mature gilts (Large White × Polish Landrace) at an abattoir (ethical approval not required). Animals were euthanized per European Legislation (EFSA, AHAW/04-027). Small (2–4 mm), medium (4–6 mm), and large (8–12 mm) follicles were isolated on days 4–6, 10–12, and 16–18 of the estrous cycle (n = 6) following morphological assessment [15]. Entire ovarian follicles were dissected for mRNA and protein expression analyses. After follicle puncture/opening, FF together with the cumulus-oocyte complex (COCs) was released and removed; therefore, the analyzed follicular tissue mainly consisted of Gc, theca cells, and surrounding stromal tissue. Follicles were frozen in liquid nitrogen for mRNA (*ENHO, GPR19*) or homogenized in lysis buffer for protein expression studies. Lysates were centrifuged (15 000 g, 4°C, 30 min), and protein content was measured (Bradford method). The FF was aspirated, centrifuged (5000 g, 4°C, 10 min), and frozen. Additionally, medium follicles from mature pigs (n = 3) were fixed, dehydrated, embedded in paraplast, and analyzed for adropin and GPR19 by immunolocalization.

### Granulosa cells culture and experimental procedure

Granulosa cells cultures were prepared from medium follicles (days 10–12 of the estrous) as described [16]. Medium follicles were selected because they showed relatively high expression of both adropin and GPR19 in our analyses (Figure  1) and represent growing antral follicles before the periovulatory hormonal surge [17]. The Gc were scraped from the follicular wall, rinsed with PBS, treated with DNase I (500 U, 1 min), washed in M199, and resuspended in M199 with 10% FBS. Cells viability was 90% (trypan blue test). Cells (5 × 10 [4]/well) were seeded in 96-well plates and preincubated for 24 h. Medium was then replaced with M199 containing 1% FBS. Cultures were maintained at 37°C in 5% CO₂/95% air.

**Figure 1:**
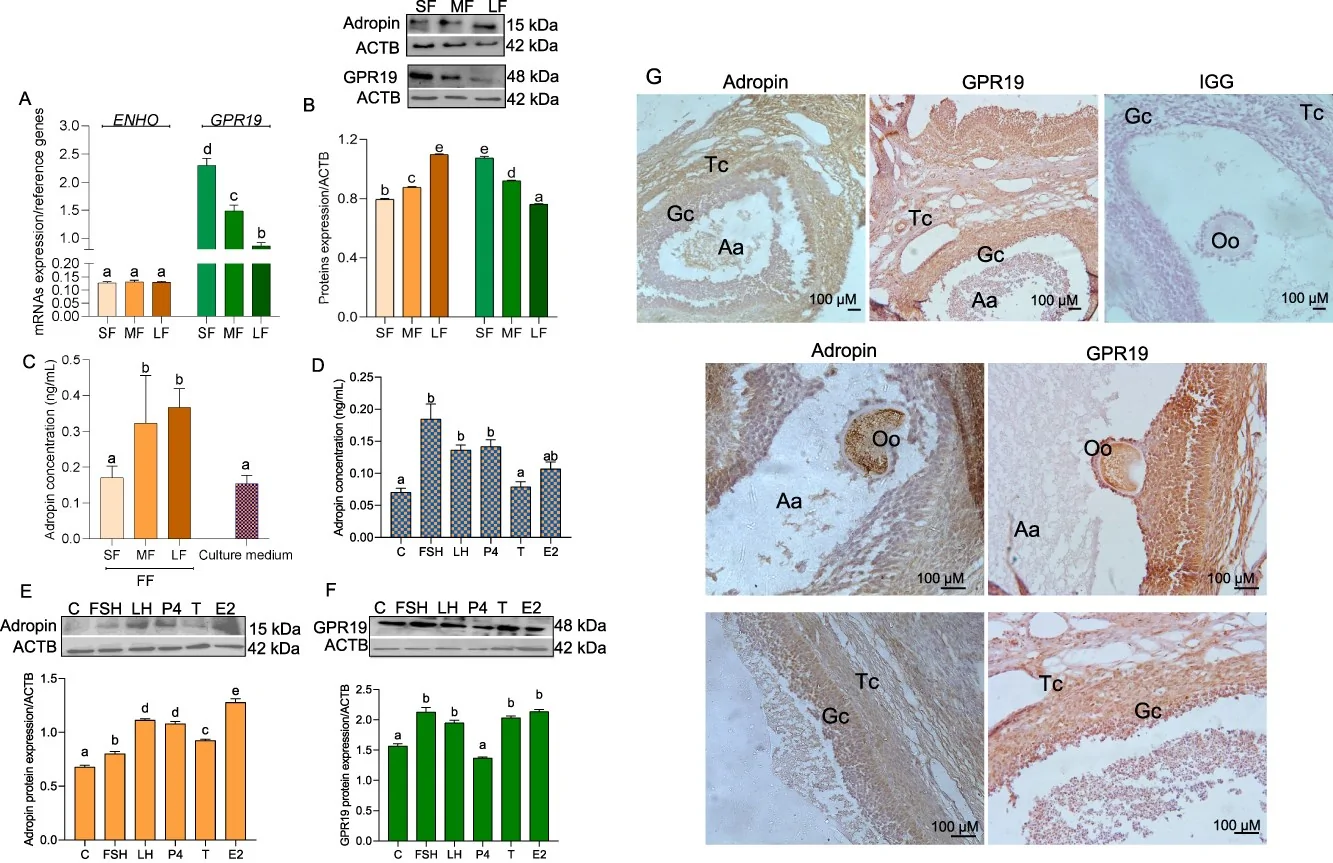
Comparison of Energy Homeostasis Associated (ENHO)/adropin and G-protein coupled receptor 19 (GPR19) mRNA (A) and protein (B) level in porcine ovarian follicle during estrous cycle, its concentration in follicular fluid (FF) (n = 5) (C), its expression regulation (D–F) as well as it’s immunolocalization (G) (n = 3). Representative blots with densitometry and immunohistochemistry photos are shown (scale bar 100 μM). Gene expression level was normalized to cyclophilin A (*PPIA*), glyceraldehyde 3-phosphate dehydrogenase (*GAPDH*) and β-actin (*ACTB*) geometric mean, while protein level was normalized to ACTB. Data are plotted as the mean ± SEM. One-way ANOVA, followed by post hoc tests was used for statistical analysis. Significance between groups is indicated by different letters (*P < 0.05*). SF—small follicle (days 2–3 of estrous cycle), MF—medium follicle (days 10–12 of estrous cycle), LF—large follicle (days 16–18 of estrous cycle), Gc—granulosa cells, Tc—theca cells, Aa—antrum, Oo—oocyte, FSH—follicle stimulating hormone, LH—luteinizing hormone, P4—progesterone, T—testosterone, E2—estradiol.

*Experiment 1:* To determine the regulation of adropin and GPR19 levels, Gc were incubated for 48 h in M199 medium containing 1% FBS [v/v] as a control, or supplemented with follicle stimulating hormone (FSH, 100 ng/mL), luteinizing hormone (LH, 100 ng/mL), P4, testosterone (T), or E2 at 10^−7^ M. The doses of gonadotropin and steroid were chosen based on our previous studies [18]. After incubation, Gc were washed in PBS and boiled in Laemmli buffer for 4 min to quantify protein expression of adropin and GPR19, while the culture medium was collected to analyze concentration of adropin.

*Experiment 2:* To determine effects of adropin on cells viability and proliferation, Gc (5 × 10 [4]/well) were incubated for 48 h in M199 containing 1% FBS [v/v] as a control, or with adropin at a doses of 0.1, 1, 10, 100 nM. The adropin doses were chosen based on the literature [19] and its concentration in FF (~0.4 ng/mL); 0.1 nM represented a physiologically relevant dose, whereas higher doses were used to evaluate broader biological and potential pathophysiological effects. Subsequently, the alamarBlue reagent (no. DAL1100, Invitrogen) was added for 2 h to evaluate Gc viability as described previously [20] or bromodeoxyuridine (BrdU) test was performed to analyze Gc proliferation [21].

*Experiment 3:* To study the effect of adropin on the expression of proliferation (proliferating cells nuclear antigen (PCNA), cyclin D1), apoptosis (caspase 3, BAX, BCL-2, p53), or autophagy (beclin-1, Lysosome-Associated Membrane Protein 1 (LAMP1), Microtubule-Associated Protein 1 Light Chain 3 (LC3)) markers, as well as aromatase (CYP19A1) expression, Gc (5 × 10 [4]/well) were incubated for 48 h in M199 containing 1% FBS [v/v] alone or with 0.1–100 nM adropin. Then Gc were washed in PBS and stored at −70°C to quantify mRNA levels or boiled in Laemmli buffer for 4 min to quantify protein expression. Additionally Caspase-Glo 3/7 reagent was added for 1.5 h to evaluate caspase-3/−7 activity.

*Experiment 4:* To investigate the effect of adropin on E2 and P4 secretion, Gc (5 × 10 [4]/well) were incubated for 48 h in M199 containing 1% FBS [v/v] alone or with 0.1, 1, 10, 100 nM of adropin. The culture medium was collected and stored at −20°C to measure the E2 and P4 concentration by ELISA.

*Experiment 5:* For cell cycle analysis, Gc (1 000 000 /well of 6-well plate) were incubated with 10 nM of adropin for 48 h and then fixed with 70% cold ethanol at 4°C for 60 min and stored at −20°C for flow cytometry analysis as described previously [22]. Dose of adropin was chosen based on previous experiments.

*Experiment 6:* To measure the effect of adropin on the phosphorylation of several kinases (pMAP3/1 (ERK1/2), MAP3/1, pAKT, AKT, pPKA and PKA), Gc (5 × 10 [4]/well) were incubated with 10 nM of adropin for 1, 5, 15, 30, and 45 min. Then collected Gc were boiled in a Laemmli buffer for 4 min to evaluate protein expression.

*Experiment 7:* To assess the involvement of MAP3/1, AKT and PKA in the effect of adropin on Gc viability, proliferation and E2 secretion, Gc (5 × 10 [4]/well) cultured in M199 supplemented with 1% FBS [v/v] were pre-treated for 1 h with a pharmacological inhibitors of MAP3/1 (PD98059, at 25 μM), AKT (LY294002 at 5 μM) or PKA (KT570 at 50 ng/mL). The doses of inhibitors were chosen based on preliminary experiments and the literature [20, 22]. Subsequently, 10 nM of adropin was added for the next 48 h. The alamarBlue reagent was added to evaluate Gc viability, BrdU assay was performed to analyze proliferation or culture medium was collected to measure the E2 concentration.

### Oocytes collection and experimental procedure

Ovaries were collected from prepubertal gilts (4–5 months old). Follicles (3–6 mm; 15/ovary) were aspirated from sow ovaries (~100/experiment) using a 12-gauge needle [23]. Part of COCs and oocytes (n = 3) were fixed for immunofluorescence of adropin and GPR19. In vitro maturation was performed as described [24]. Only COCs with ≥3 cumulus layers and uniform cytoplasm were selected. Groups of 12–15 COCs were cultured in 100 μL maturation medium under mineral oil. Cultures were maintained at 39°C in 5% CO₂/95% O₂. After maturation, oocytes were denuded using 0.5% hyaluronidase. Cumulus cells were obtained by centrifugation (1000 g, 5 min).

*Experiment 1:* To evaluate adropin and GPR19 expression before and after in vitro maturation, COCs were cultured in TCM199 maturation medium for 44 h. Afterward, cumulus cells were removed. Denuded oocytes (n = 10/analysis) and cumulus cells (n = 10/analysis) were frozen at −20°C for mRNA and protein expression assessment. The experiment was repeated four times (n = 4)*.*

*Experiment 2:* To investigate the impact on in vitro maturation of oocytes, COCs were cultured in TCM199 medium with or without adropin (10 nM). After 44 h of culture, cumulus cells were separated from COCs. Denuded oocytes (n = 15/group) were fixed in 4% paraformaldehyde for nuclear maturation assessment using DAPI (oocytes with first polar body were assessed as mature), fixed in 4% paraformaldehyde and stained with NileRed to measure lipid droplet content or frozen in −70°C for adipose differentiation-related protein (*ADRP*) and adipose triglyceride lipase (*ATGL*) mRNA expression analysis. The culture medium was collected for P4 concentration measurement. The experiments were repeated four times (n = 4).

### RNA isolation and real-time PCR

Total RNA was isolated using TRIzol reagent according to the manufacturer’s protocol as described previously [25] or by Cell to CT reagent (Applied Biosystem). Reverse transcription and real-time PCR analyses were performed using SYBR Green or TaqMan assays depending on the target samples. SYBR Green chemistry was used to evaluate *ENHO* and *GPR19* expression in ovarian follicles and COCs. The primer sequences were as follows: adropin (*ENHO*) (forward 5′-CTCGTAGGCTTCTTGCTGCT-3′ and reverse 5′-TTCCGAGAGGGAGTCGATGT-3′), *GPR19* (forward 5′-CACAAGCAGTGGATTTGGGC-3′ and reverse 5′-CCACCAGGAAAGCTTGGGAA-3′), *PPIA* (forward 5′-CACAAACGGTTCCCAGTTTT-3′ and reverse 5′-TGTCCACAGTCAGCAATGGT-3′), beta actin (*ACTB)* (forward 5′-CACGCCATCCTGCGTCTGGA-3′ and reverse 5′-AGCACCGTGTTGGCGTAGAG-3′) and *GAPDH* (forward 5′-GCACCGTCAAGGCTGAGAAC-3′ and reverse 5′-ATGGTGGTGAAGACGCCAGT-3′). TaqMan gene expression assays (Applied Biosystems) were used to quantify mRNA expression of proliferation, apoptosis, autophagy markers, *CYP19A1, ADRP*, and *ATGL*, based on a previously described procedure [20]. Amplifications were performed using the StepOnePlus system (Applied Biosystems) according to the manufacturer’s instructions and TaqMan probes listed in Table 1. Relative gene expression was normalized to *PPIA, GAPDH*, and *ACTB* according to Vandesompele et al. [26] and our previous experience with porcine Gc [27]. Ct values were converted into relative quantities using assay-specific amplification efficiencies. For each sample, the relative quantity of the target gene was normalized by division by the geometric mean of the relative quantities of reference genes, according to Vandesompele et al. [26] and Hellemans et al. [28]. No additional calibration to the control group was performed.

**Table 1:** TaqMan-specific primers

Gene symbol	Gene name	Assay ID	Reference sequence
*CCND1*	cyclin D1	Ss06875967	XM_021082686.1
*PCNA*	proliferating cell nuclear antigen	Ss03377029	NM_001291925.1
*CYP19A1*	cytochrome P450 family 19 subfamily A member 1	Ss03384876	NM_214429.1
*CASP3*	caspase 3	Ss03382792	NM_214131.1
*BAX*	BCL2 associated X, apoptosis regulator	Ss03375842	XM_003127290.5
*BCL2*	BCL2 apoptosis regulator	Ss03375167	XM_021099593.1
*P53 (TP53)*	tumor protein p53	Ss04248637	NM_213824.3
*BCN1*	beclin	Ss03380214	NM_001044530.1
*LAMP1*	lysosomal associated membrane protein 1	Ss03378990	NM_001011507.1
*LC3 (MAP1LC3A)*	microtubule-associated protein 1 light chain 3	Ss04246430	NM_001170827.1
*ADRP*	adipose differentiation-related protein	Ss03394357	NM_214200.2
*ATGL*	adipose triglyceride lipase	Ss03385996	NM_001098605.1
*PPIA*	cyclophilin A	Ss03394782	NM_214353.1
*GAPDH*	glyceraldehyde 3-phosphate dehydrogenase	Ss03375629	NM_001206359.1
*ACTB*	actin	Ss03376563	XM_003124280.5

### Western blot

Electrophoresis, transfer, and Western blotting were performed as described [20]. Briefly, 30–50 μg of protein were analyzed by Western blot. Details of primary and secondary antibodies are provided (Table 2). ACTB or tubulin (TUBA) antibodies were used as loading controls. Signals were detected by chemiluminescence (WesternBright Quantum, HRP) and visualized using the Chemidoc XRS+ system (BioRad). Band intensity was quantified with a densitometer and ImageJ (US NIH). Representative blots and densitometric data are shown. Full blots showed in Supplemental Figure S1.

**Table 2:** Antibodies used in Western blot

Antibody	Host/Target	Dilution	RRID	Supplier and product no.
Adropin	Rabbit	1:500	AB_2718635	Thermo Fisher Scientific, PA5-72781
GPR19	RabbitRabbit	1:2001:200	--	BosterBio, A14590BosterBio, A14590
Cyclin D	Rabbit	1:1000	AB_2750906	Abcam, ab134175
CYP19A1	Rabbit	1:200	AB_2088676	Thermo Fisher Scientific, PA1-21398
pERK1/2	Rabbit	1:1000	AB_331646	Cell Signaling Technology, 9101S
tERK1/2	Rabbit	1:1000	AB_330744	Cell Signaling Technology, 9102S
pAKT	Rabbit	1:1000	AB_329825	Cell Signaling Technology, 9271S
tAKT	Rabbit	1:1000	AB_329827	Cell Signaling Technology, 9272S
pPKA	Rabbit	1:1000	AB_305132	Abcam, ab5815
tPKA	Rabbit	1:1000	AB_3665519	Abcam, ab187515
Caspase 3	Rabbit	1:1000	AB_331439	Cell Signaling Technology, 9662S
PCNA	Mouse	1:250	AB_2533016	Thermo Fisher Scientific, 13-3900
Beclin1	Rabbit	1:300	–	Cell Signaling Technology, D40C5
LAMP1	Rabbit	1:200	AB_2687579	Cell Signaling Technology, 9091
LC3	Rabbit	1:250	AB_2617131	Cell Signaling Technology, 12,741
β-actin	Mouse	1:5000	AB_476743	Sigma-Aldrich, A5316
Anti-rabbit	Goat	1:1000	AB_2099233	Cell Signaling Technology, 7074
Anti-mouse	Horse	1:1000	AB_330924	Cell Signaling Technology, 7076

### Immunohistochemistry

Five μm paraffin-embedded ovarian follicle sections were processed. Nonspecific binding was blocked with 5% [w/v] goat serum (30 min), incubated with primary antibodies against adropin or GPR19 (1:50 [v/v], Table 2). Biotinylated secondary IgG (1:300 [v/v]), an avidin-biotin-HRP complex (1:100 [v/v]), and DAB chromogen (1:400 [v/v], Sigma–Aldrich) were applied. Sections were counterstained with hematoxylin, dehydrated, and mounted in DPX. Negative controls used non-immune rabbit IgG. Imaging was done using a Nikon Eclipse Ni-U microscope with a DS-Fi1-U3 camera and Plan Apo λ 20×/0.75 objective.

### Immunofluorescence

COCs and oocytes were fixed in 4% paraformaldehyde [v/v], rinsed in PBS, and incubated in 0.1 M glycine/PBS (15 min). Permeabilization was done with 0.15% Triton X-100 [v/v] in PBS containing 1% BSA [w/v] (15 min), followed by blocking in 2% BSA/PBS [w/v] (15 min). Samples were then incubated for 1 h with anti-adropin or anti-GPR19 antibodies (1:50 in 1% BSA/PBS). After washing, Alexa Fluor 488-conjugated anti-rabbit secondary antibody (1:500; A11008, Invitrogen) was applied for 1 h in the dark. Negative controls omitted primary antibodies. Fluorescent imaging was performed using a Zeiss Axioplan microscope.

### NileRed staining

To visualize lipid droplets, oocytes were stained with NileRed (Sigma–Aldrich). Oocytes were fixed in 4% paraformaldehyde [v/v] in PBS (30 min, RT), then washed 3× in PBS (5 min each). Permeabilization was done with 0.1% Triton X-100 [v/v] in PBS (10 min), followed by PBS washes. NileRed stock (1 mg/mL in DMSO) was diluted to 1 μg/mL in PBS before use. Oocytes were incubated for 20 min in the dark, then washed 3× in PBS. Imaging was performed with a Zeiss Axioplan microscope using filters for NileRed (excitation: 543–552 nm; emission: 590–620 nm). Lipid droplet content was analyzed using ImageJ.

### ELISA

To measure adropin concentration in FF and culture medium porcine ELISA kit was used (QnD, no. EIA07297). The limit of assay sensitivity was 0.156 ng/mL. The inter- and intra-experimental coefficients of variation were <12% and <8%. To determine the E2 and P4 concentration in culture medium ELISA kit was used (DRG Instruments GmbH, EIA-1561 and EIA-2693). The limit of assay sensitivity was 9.714 pg/mL, 0.045 ng/mL. The inter- and intra-experimental coefficients of variation were <9.39% and <6.81%, <9.96% and <6.99%, respectively. Samples were run in duplicate within the same assay. Absorbance was measured at the 405 nm wavelength using a Varioskan LUX reader (Thermo Fisher Scientific).

### AlamarBlue

To assess Gc viability the alamarBlue reagent (Invitrogen) was aseptically added to each well at a volume equivalent to 10% [v/v] of the total incubation volume. Following a 2 h incubation, fluorescence was measured at 530 and 590 nm using a Varioskan Lux reader (ThermoFisher Scientific).

### Bromodeoxyuridine

Cells proliferation was assessed using the BrdU assay (no. 11647229001, Roche Diagnostics). Bromodeoxyuridine was added to each well at 20% of the culture medium volume and the assay was performed according to the manufacturer’s instructions. Absorbance was measured at 450 nm using a Varioskan Lux.

### Flow cytometry

Cells pellets were rinsed and resuspended in 1 mL PBS, treated with 50 μg RNase A and 30 μg propidium iodide (PI). After 30-min samples were centrifuged and resuspended. PI-stained, RNA-depleted cells were analyzed on a CytoFlex flow cytometer by measuring DNA fluorescence (λ = 690 nm). Doublets were excluded using area vs. height pulse analysis, and at least 10^4^ cells per sample were acquired. DNA ploidy and cell cycle phases were assessed using Kaluza software (v2.2.1).

### Caspase-Glo 3/7 assay

Caspase-3/7 activity was determined using the Caspase-Glo® 3/7 Assay (Promega, no. G8090), according to the manufacturer’s instructions. The assay is based on cell lysis followed by cleavage of a luminogenic substrate by active caspases-3 and -7, resulting in the release of aminoluciferin. In the presence of luciferase, aminoluciferin generates a luminescent signal proportional to caspase-3/7 enzymatic activity. The Caspase-Glo reagent was added directly to the culture medium at a 1:1 ratio (v/v). After 1.5 h incubation at room temperature, luminescence was recorded using Varioskan Lux.

### Statistical analysis

Data are presented as the means ± SEM. Distribution of normality was checked by the Shapiro–Wilk test. Statistical analysis was carried out using T-test or one-way or two-way ANOVA, followed by the Tukey test (PRISM software version 5; GraphPad). Statistical significance is indicated by different letters (*P < 0.05*).

## Results

### Expression of *ENHO*/adropin and GPR19 in the porcine ovarian follicle during the estrous cycle

The aim of this experiment was to assess the expression of *ENHO*/adropin and its receptor GPR19 in porcine ovarian follicles across the estrous cycle and to determine whether their levels were hormonally regulated. In follicles *ENHO* mRNA remained stable across the estrous cycle, while GPR19 mRNA and protein levels declined (Figure  1A, *P < 0.05*). Adropin protein was highest in large follicle. This pattern was consistent with adropin levels in FF, which mirrored its expression in ovarian follicles (0.171 ± 0.013, 0.323 ± 0.055, 0.368 ± 0.022 ng/mL, respectively). Adropin was also detected in the culture medium from medium follicle-derived Gc, at levels similar to adropin level in FF collected from small follicles (0.154 ± 0.010 ng/mL) (Figure  1B and C, *P* *< 0.05*). FSH, LH, and P4 increased adropin secretion into the culture medium. All investigated gonadotropins and steroids elevated adropin and GPR19 levels in Gc, except P4 for GPR19, which had no effect (Figure  1D–F, *P* *< 0.05*). Immunolocalization confirmed adropin and GPR19 in the Gc and theca cells of medium follicle both in the cytoplasm and nuclei (Figure  1G). In summary, adropin and its receptor are present in the ovarian follicle, and their expression levels vary depending on the phase of the estrous cycle and are regulated by gonadotropins and steroids.

### Effect of adropin on granulosa cells viability, proliferation, proliferating cells nuclear antigen and cyclin D1 expression

The aim of this experiment was to determine whether adropin affected Gc viability and proliferation. Adropin only at 10 nM significantly reduced Gc viability (Figure  2A) and inhibited proliferation at all tested doses (0.1–100 nM) (Figure  2B) (*P < 0.05*). It also reduces cells number in S phases (10 nM) (Figure  2C, *P < 0.05*). These effects were supported by decreased PCNA mRNA (1–100 nM; Figure  2D) and protein levels (all doses; Figure  2E), as well as downregulation of cyclin D1 (*CCND1*) mRNA (0.1–10 nM; Figure  2F) and protein (0.1–100 nM; Figure  2G) (*P < 0.05*). Together, these data indicated that adropin exerted a suppressive effect on Gc proliferation.

**Figure 2:**
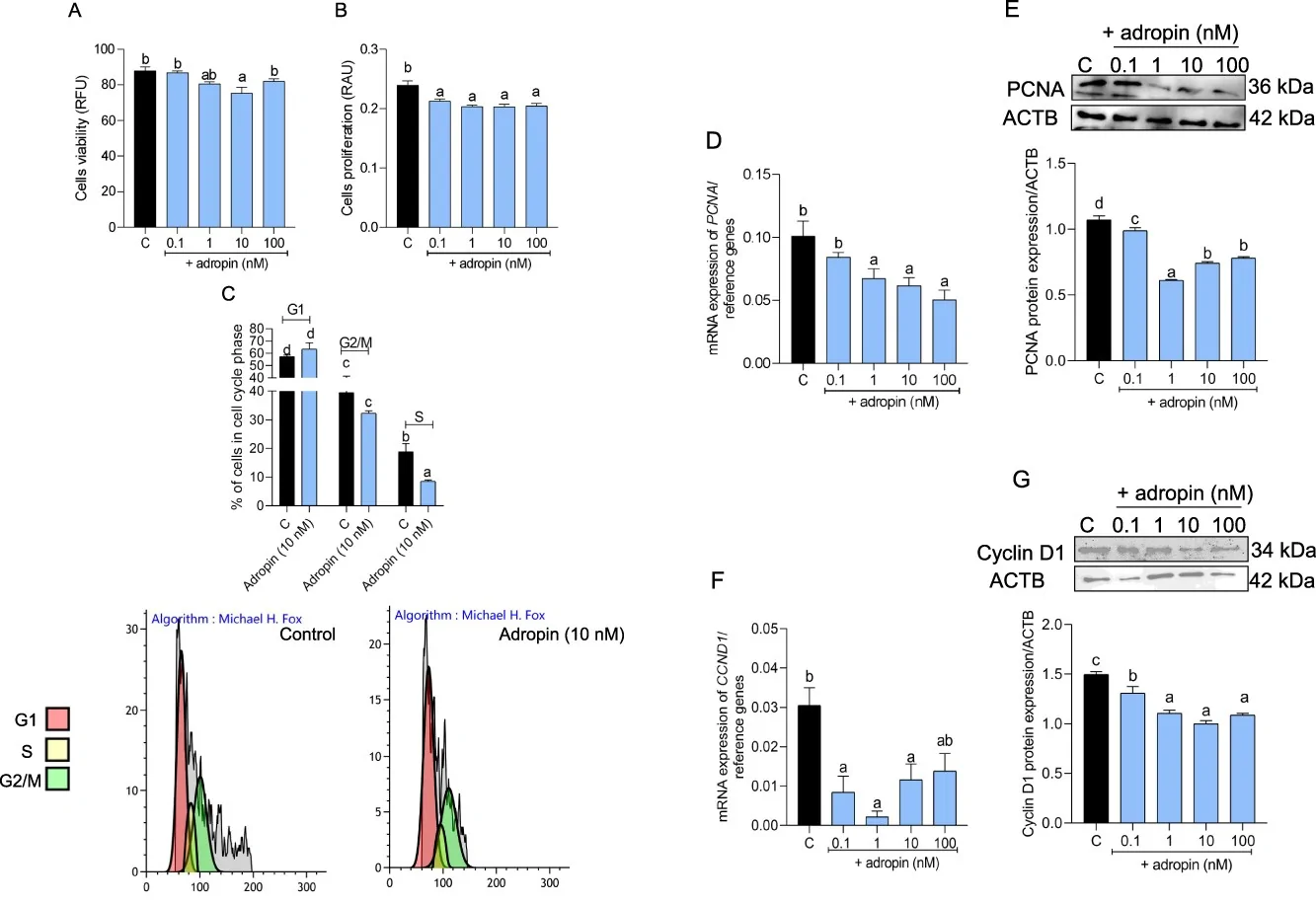
Effect of adropin on porcine granulosa cells viability *via* alamarBlue (A), proliferation *via* BrdU (B), cell cycle progression (C) proliferating cells nuclear antigen (PCNA) and cyclin D1 (*CCND1*) mRNA (D, F) and protein (E, G) expression. Representative blots and densitometry are shown. Gene expression level was normalized to cyclophilin A (*PPIA*) and Glyceraldehyde-3-phosphate dehydrogenase (*GAPDH*), while protein to β-actin (*ACTB*). Data are plotted as the mean ± SEM. One-way or two-way (cell cycle analysis) ANOVA test was used for statistical analyses. Significance between groups is indicated by different letters at *P < 0.05* (n = 5). C: control, RFU: relative fluorescence units, RFA: relative absorbance units.

### Effect of adropin on E2 and P4 secretion and CYP19A1 expression

The aim of this experiment was to determine whether adropin modulated E2 and P4 production and CYP19A1 expression in Gc. Adropin at doses 0.1, 10, and 100 nM significantly inhibited E2 secretion by Gc at 240.025 ± 18.808 pg/mL, 300.1 ± 12.694 pg/mL, and 266.6 ± 8.71 pg/mL, respectively vs. 383.32 ± 6.25 pg/mL in control (Figure  3A, *P < 0.05*). Also, we observed that adropin at doses 0.1 and 1 nM downregulated *CYP19A1* mRNA level (Figure  3B, *P < 0.05*), while at all investigated doses CYP19A1 protein expression (Figure  3C, *P < 0.05*). Finally, adropin increased P4 secretion by Gc at doses 0.1 and 100 nM by 6.80 ± 0.26 and 6.37 ± 0.27 ng/mL compared with 4.88 ± 0.17 ng/mL in control (Figure  3D*, P < 0.05*). These findings indicated that adropin exerted an regulatory effect on Gc steroidogenesis.

**Figure 3:**
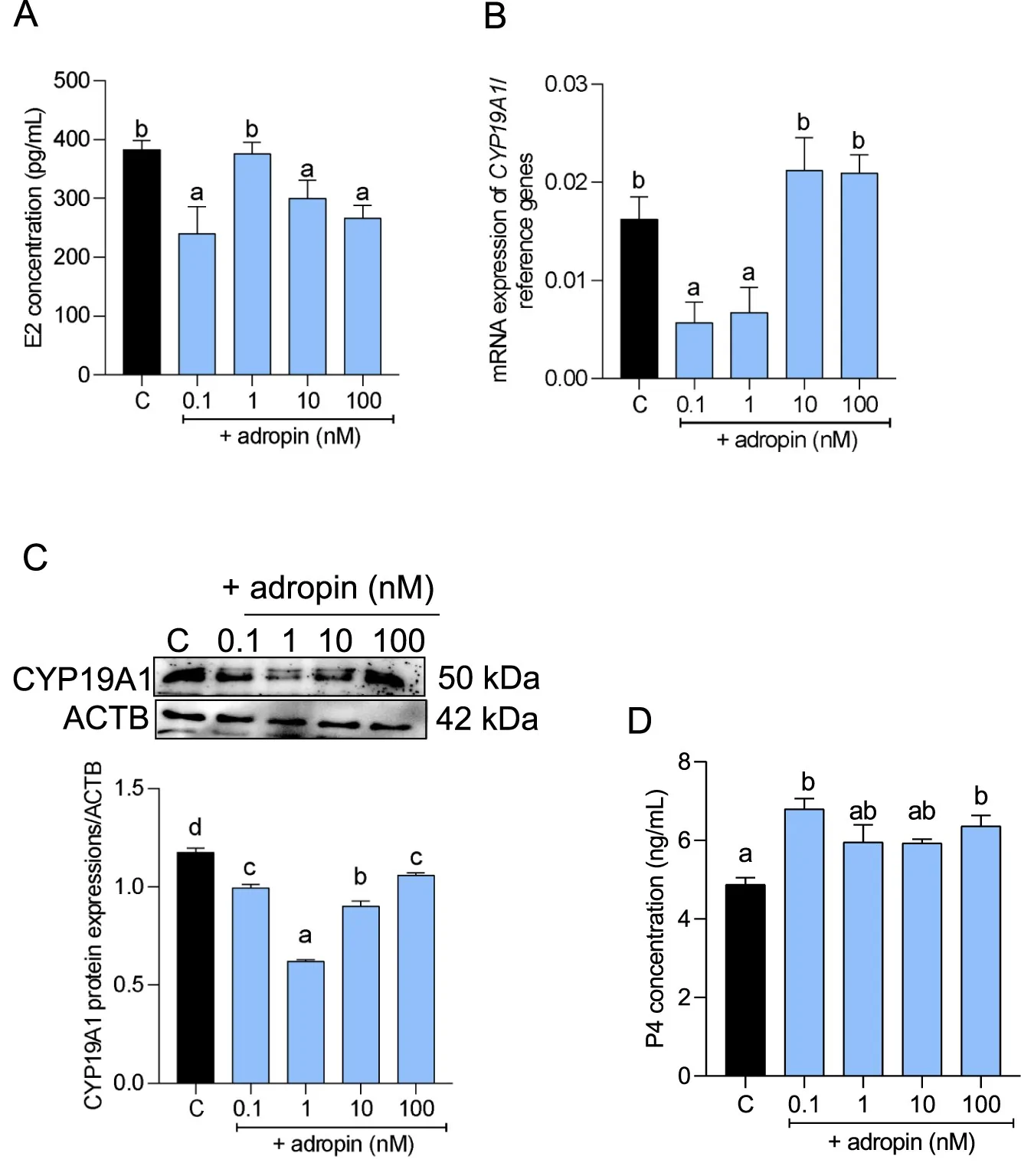
Effect of adropin on porcine granulosa cells estradiol (E2) secretion (A) and aromatase (CYP19A1) mRNA (B) and protein (C) expression, as well as progesterone (P4) secretion (D). Representative blots and densitometry are shown. Gene expression level was normalized to cyclophilin A (*PPIA*) and Glyceraldehyde-3-phosphate dehydrogenase (*GAPDH*), while protein to β-actin (ACTB). Data are plotted as the mean ± SEM. One-way ANOVA test was used for statistical analyses. Significance between groups is indicated by different letters at *P < 0.05* (n = 5). C: control.

### Effect of adropin on granulosa cells apoptosis and autophagy markers level

The aim of this experiment was to assess whether adropin influenced apoptotic and autophagy-related signaling in Gc. As shown in Figure  4, adropin did not alter caspase-3 (*CASP3*) mRNA (Figure  4A, *P* *> 0.05*) but significantly increased cleaved caspase-3 protein at all doses (Figure  4B, *P* *< 0.05*) and caspase 3/7 activity at dose 10 nm (Figure  4C, *P* *< 0.05*). Additionally adropin increased *BAX* and *BCL2* mRNA expression at dose 10 nM with no effect on *p53* mRNA (Figure  4D, *P* *< 0.05*). At only 10 nM, it reduced *BECN1* mRNA (Figure  4E), and all doses lowered beclin-1 protein (Figure  4F, *P* *< 0.05*). *LC3* mRNA decreased at 0.1 and 100 nM (Figure  4G, *P* *< 0.05*), with no change in LC3 protein (Figure  4H, *P* > 0.05). *LAMP1* mRNA was unaffected (Figure  4I, *P* *> 0.05*), though protein levels dropped at 0.1 and 1 nM (Figure  4J, *P* *< 0.05*). Collectively, these results indicated that adropin activated apoptotic signaling while suppressing autophagy-related pathways in Gc.

**Figure 4:**
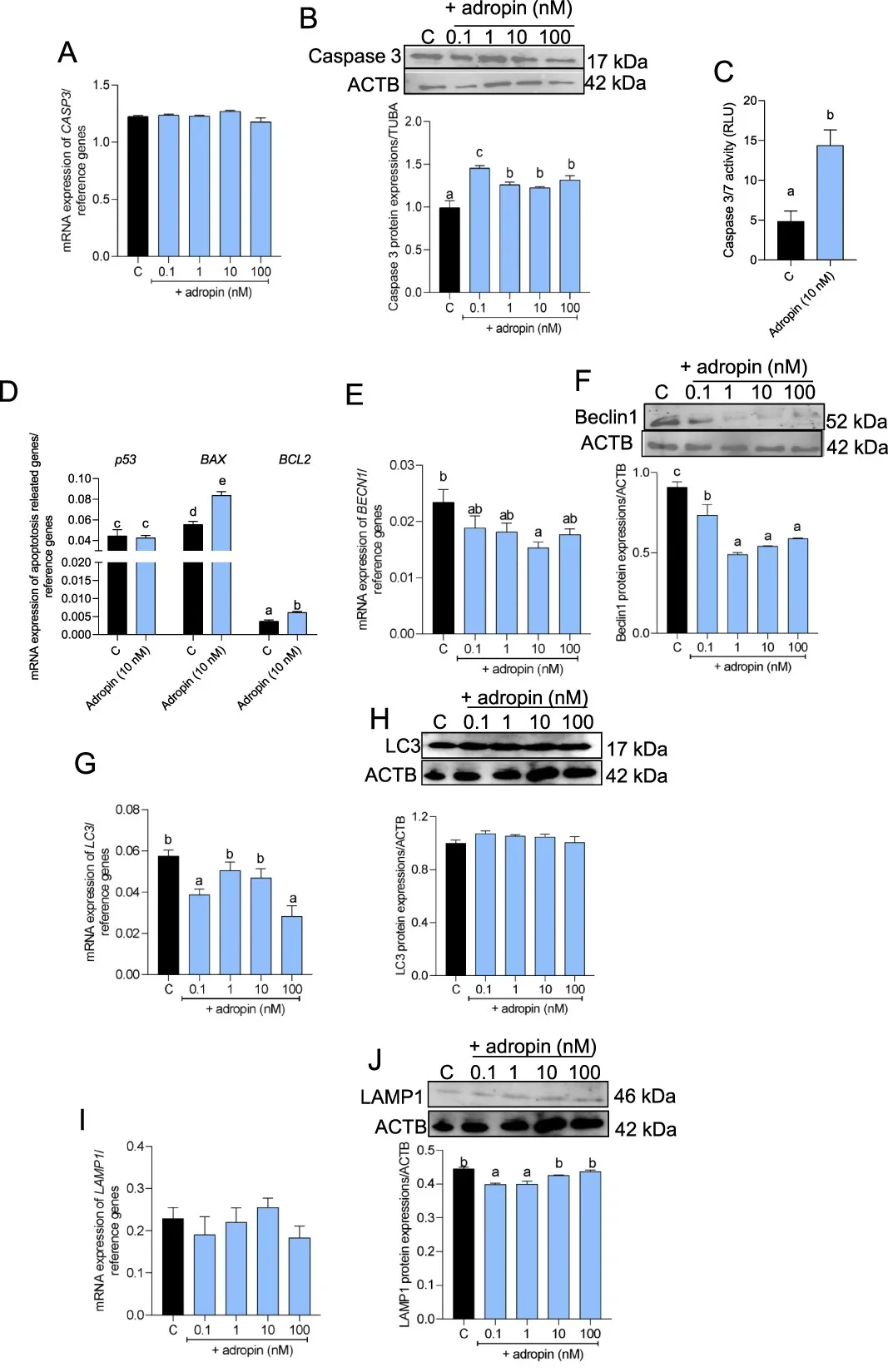
Effect of adropin on apoptosis and autophagy markers in porcine granulosa cells measured by caspase 3 (*CASP3*), p53, BAX, BCL2, Beclin1 (*BECN1*), Microtubule-associated protein 1A/1B-light chain 3 (LC3) and Lysosome-Associated Membrane Protein 1 (LAMP1) mRNA (A, D, E, G, I) and protein (B, F, H, J) expression as well as caspase 3/7 activity (C). Representative blots and densitometry are shown. Gene expression level was normalized to cyclophilin A (*PPIA*) and Glyceraldehyde-3-phosphate dehydrogenase (*GAPDH*), while protein to β-actin (ACTB). Data are plotted as the mean ± SEM. One-way ANOVA test was used for statistical analyses. Significance between groups is indicated by different letters at *P < 0.05* (n = 5). C: control.

### Effect of adropin on the phosphorylation of MAP3/1, AKT, and PKA kinases pathways and their involvement in adropin action on granulosa cells viability, proliferation, and E2 secretion

The aim of this experiment was to determine whether adropin activated MAP3/1 (ERK1/2), AKT, and PKA signaling pathways in Gc, and whether these pathways mediated its effects on Gc function. Adropin (10 nM) increased phosphorylation of AKT kinase after 5, 15, 30, and 45 min of in vitro culture; phosphorylation of ERK1/2 after 15, 30, and 45 min; and phosphorylation of PKA after 1, 15, 30, and 45 min (Figure  5A, *P* < 0.05). We observed that the negative effect of adropin (10 nM) on Gc viability was reversed to control following pharmacological inhibition of the AKT pathway; the effect on proliferation was reversed after inhibition of ERK1/2 (Figure  5B and C, *P* < 0.05), while its negative effect on E2 synthesis was reversed after pharmacological inhibition of the PKA pathway (Figure  5C, *P* < 0.05). Together, these data demonstrated that AKT, ERK1/2, and PKA pathways mediated the functional actions of adropin in porcine Gc.

**Figure 5:**
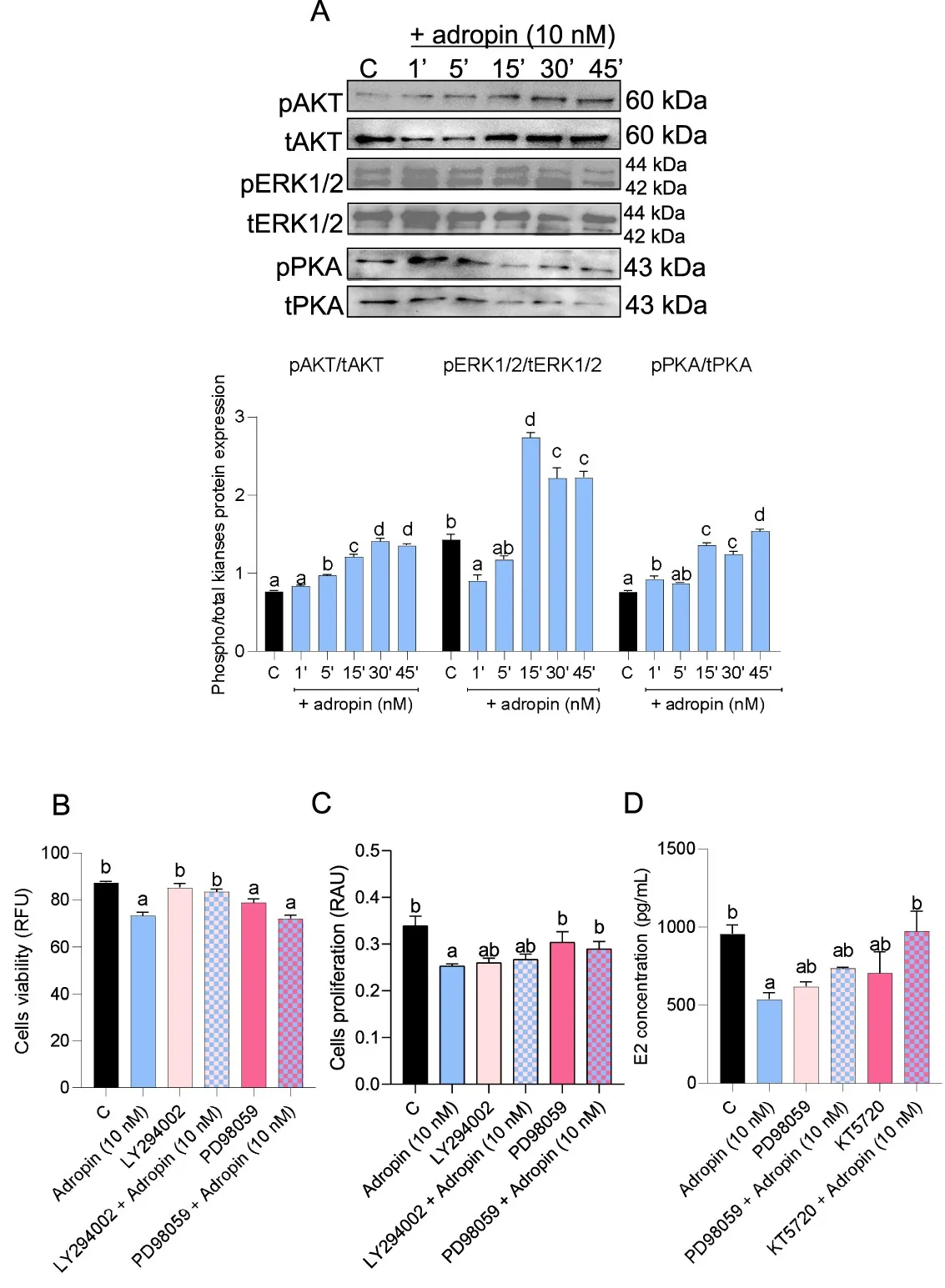
Effect of adropin on phosphorylation of mitogen activated kinase (ERK1/2; MAP3/1), protein kinase B (AKT) and protein kinase A (PKA) (A) and their involvement in adropin mediated action on porcine granulosa cells viability (B), proliferation (C) and E2 synthesis (D). Representative blots and densitometry are shown. Protein levels were normalized to the total form of each kinase. Data are plotted as the mean ± SEM. One-way ANOVA, followed by post hoc tests was used for statistical analysis. Significance between groups is indicated by different letters at *P < 0.05* (n = 5). C: control; RFU: relative fluorescence units, PD98059: MAP3/1 inhibitor, LY294002: AKT inhibitor, KT5720: PKA inhibitor.

### Effect of adropin on the oocytes maturation

The aim of this experiment was to investigate whether adropin and its receptor GPR19 were involved in porcine oocyte maturation. Following maturation, *ENHO* mRNA expression significantly increased in cumulus cells but remained unchanged in oocytes, whereas adropin protein expression increased in both oocytes and cumulus cells (Figure  6A and B, *P* < 0.05). Whereas GPR19 increased only in cumulus cells after maturation at both mRNA and protein level, whereas no statistically significant changes were observed in oocytes, although *GPR19* mRNA showed a non-significant downward trend (Figure  6C and D, *P < 0.05*). Immunolocalization of both adropin and GPR19 was observed in oocytes and cumulus cells (Figure  6E). Additionally, adropin enhanced the percentage of oocytes reaching metaphase II (Figure  6F, *P* < 0.05), stimulated P4 secretion by COCs (Figure  6G, *P* < 0.05), decreased lipid droplet content (Figure  6H, *P* < 0.05), and upregulated *ATGL* mRNA expression, while down regulating *ADRP* mRNA expression (Figure  6I, *P* < 0.05). Collectively, these results demonstrated that adropin positively influenced porcine oocyte maturation and metabolic quality.

**Figure 6:**
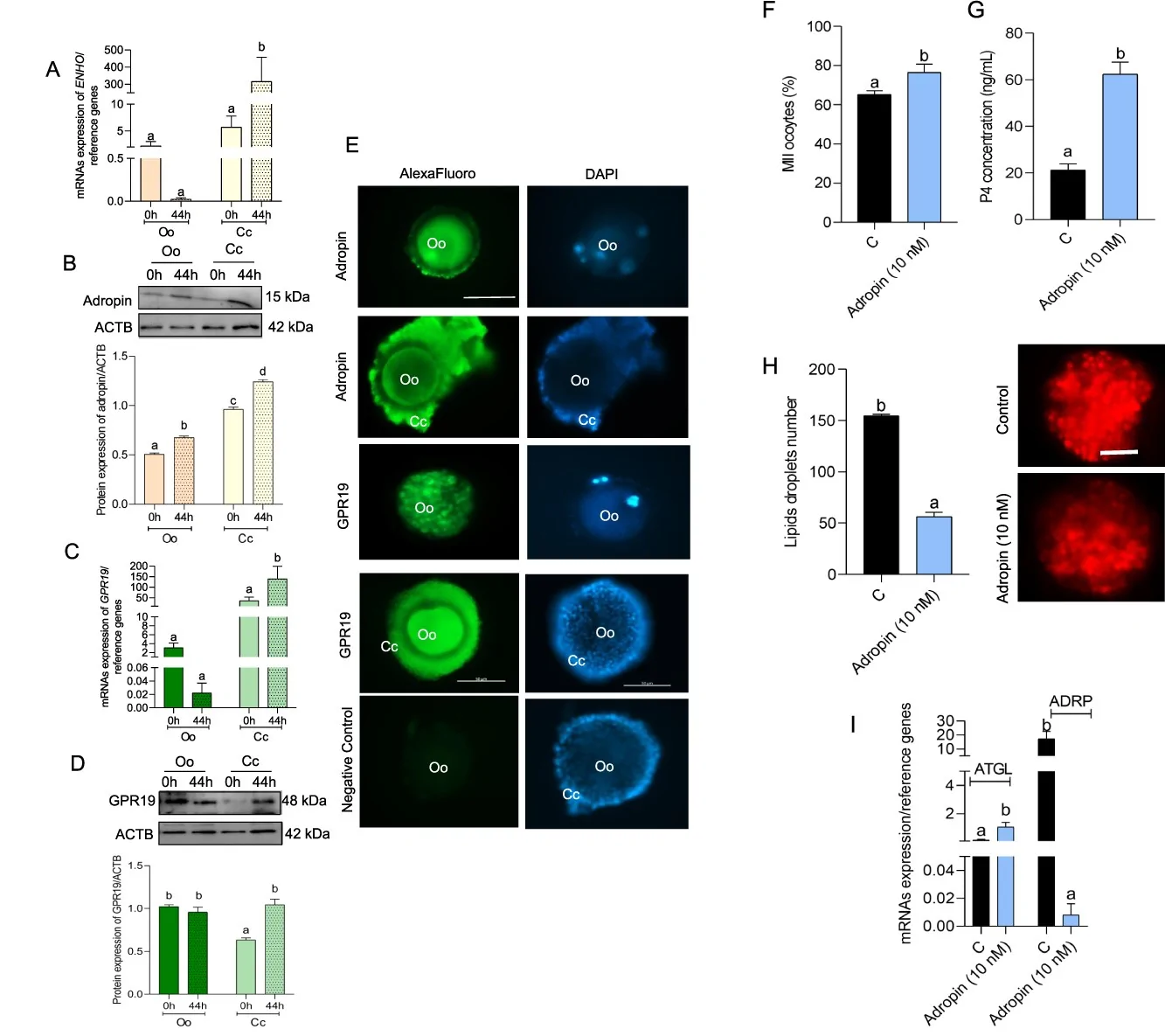
Effect of adropin on the porcine oocytes maturation. Adropin mRNA (A) protein (B), as well as G-protein coupled receptor 19 GPR19 mRNA (C) and protein (D) content in the oocytes (Oo) and cumulus cells (CCs) before (0 h) and after (44 h) oocytes maturation and its immunolocalization (E) in cumulus-oocyte complex (COCs) before maturation, as well as effect of adropin on the oocytes number in the metaphase-II (F), progesterone (P4) secretion (G), lipid droplet content (H) and adipose triglyceride lipase (*ATGL*) and adipose differentiation-related protein (*ADRP*) genes level (I) were analyzed. Representative blots with densitometry and immunofluorescence photos are shown (scale bar 50 uM). Negative control (NC), obtained by omission of the primary antibody, showed only negligible background fluorescence. Gene expression level was normalized to cyclophilin A (*PPIA*) and β-actin (*ACTB*) geometric mean, while protein level was normalized to ACTB. Data are plotted as the mean ± SEM. T-test was used for statistical analysis to compare percentage of MII oocytes, P4 concentration, lipid droplet number, and mRNA expression levels of *ATGL* and *ADRP* between C and adropin 10 nM, one-way ANOVA was used in the rest of experiments. Significance between groups is indicated by different letters (*P < 0.05*).

## Discussion

This is the first study to report adropin and GPR19 expression in porcine ovarian follicles across the estrous cycle and to examine adropin’s in vitro effects on Gc proliferation, apoptosis, autophagy, E2 secretion, cell cycle, oocyte maturation, and ERK1/2, AKT, and PKA phosphorylation. While adropin’s role in the mouse ovary has been described [13], our work broadens its functional characterization in pigs.

We found that the relative abundance of *ENHO* mRNA remained unchanged in ovarian follicles throughout the estrous, whereas adropin protein expression increased in parallel with follicular growth. Discrepancies between transcript and protein levels likely result from complex post-transcriptional regulation affecting mRNA translation and protein stability [29]. Furthermore, variations in protein levels among individual ovarian follicles are likely modulated by steroid hormones synthesized or secreted by follicles, whose concentrations fluctuate during the estrous cycle, steroids hormones and gonadotropins may directly modulates adipokines expression and secretion as described previously for vaspin [18]. We showed that gonadotropins and steroids stimulate adropin and GPR19 expression in porcine Gc and its secretion into the culture medium. This partly explains our observations in the whole ovarian follicle, where during the periovulatory period characterized by high E2 and LH, along with rising P4 levels adropin levels are the highest, whereas GPR19 expression is unchanged. Previous studies have shown that adropin expression in mouse liver is upregulated by E2, which itself increases during the progression of the estrous cycle [30]. Consistently, adropin protein level peaked during the estrus phase (comparable to large follicle phase in pigs), a stage characterized by high E2 concentrations in mice [13]. These changes are mirrored in the adropin levels detected in FF, suggesting that Gc are capable of synthesizing and modulating adropin secretion into the FF. Additionally, Gc of medium follicles cultured in vitro are capable of producing adropin at the levels comparable to those detected in FF collected from small follicle. We also observed that GPR19 mRNA and protein expression declined as the estrous cycle progressed. In mice, GPR19 protein expression increases during estrus, coinciding with elevated E2 levels [13]. While another study found increased GPR19 expression in adrenocortical carcinoma cells with high androgen production [31]. Together, these observations suggest that GPR19 expression may be hormonally regulated, but in a species- and tissue-specific manner. Alternatively, elevated adropin may induce receptor internalization, a known mechanism [32]. Finally, we demonstrated that both adropin and GPR19 are present in porcine Gc and theca cells. These findings are supported by studies in mice, which revealed strong immunoreactivity of adropin and GPR19 in Gc of large antral follicles and in the corpus luteum [13]. Another study reported intense adropin staining in theca cells, while GPR19 immunoreactivity was observed in Gc of mice [14]. Although GPR19 is a membrane-bound GPCR, its cytoplasmic localization may result from receptor internalization or trafficking [32]. Previous studies on human cortex, stomach, duodenum, colon, and prostate confirm this observation GPR19 was found in both membrane and cytoplasm with the latter representing internalized receptor form [33].

The expression of both components of the adropin signaling system—its ligand and receptor—supports the hypothesis that adropin may play a direct role in regulating porcine ovarian follicle function. Understanding adropin’s impact on Gc proliferation is key to uncovering mechanisms of follicle selection, atresia, and reproductive efficiency, as Gc proliferation is essential for folliculogenesis and oocyte development [34]. In this study, adropin reduced Gc viability (but only in non-physiological dose) and proliferation, consistent with its tissue-specific effects on cell growth. While adropin promotes proliferation in various adipocyte and adrenal cell models [31, 35], inhibits this process in vascular cells [36]. Our results show that adropin impairs cell cycle progression by reducing DNA synthesis (S phase) and mitosis (G2/M phase), accompanied by downregulation of PCNA and cyclin D—both normally elevated during these phases [37].

In addition to inhibiting proliferation, adropin also suppressed E2 secretion and CYP19A1 expression. Disruptions in E2 secretion are known to impair folliculogenesis and prevent dominant follicle selection, which are often accompanied by decreased proliferative capacity [34]. Granulosa cell proliferation is often linked to E2 synthesis modulation by adipokines and neuropeptides. Apelin and vaspin promote both processes in porcine Gc [22, 38], while spexin inhibits them in human Gc [39]. Thus, adropin may affect Gc proliferation both directly and indirectly *via* E2 suppression. Interestingly, adropin reduced CYP19A1 protein expression at all tested doses, while a significant decrease in E2 secretion was not observed at 1 nM of adropin. This lack of direct correlation suggests that adropin may modulate aromatase activity independently of its expression level. Similar discrepancies between steroidogenic enzyme activity and expression have been reported; e.g., ciprofibrate decreased Hsd3b activity in rat testes without affecting its protein or mRNA expression [40]. Although in our in vitro model adropin exerted an inhibitory effect on steroidogenesis, opposite effects have been observed in vivo. In a rat model of letrozole-induced PCOS, adropin administration elevated E2 levels [41]. It is important to note, however, that in vivo outcomes reflect the influence of systemic hormonal feedback loops between the hypothalamus, pituitary, and ovary, which are absent in vitro. Additionally, species differences may further explain the variability in adropin’s effects; in the mouse ovary, adropin was shown to increase CYP19A1 expression [42]. Interestingly, adropin significantly increased P4 secretion by Gc, suggesting that the observed reduction in cell proliferation and E2 production may be associated with a shift toward a more differentiated phenotype rather than a general suppression of Gc function [43]. However, because Gc differentiation and luteinization were not comprehensively evaluated using a broader panel of molecular and functional markers, this interpretation remains tentative and requires further confirmation.

Our findings suggest that reduced Gc proliferation may partly result from increased apoptosis, a key process in follicular atresia [44]. In this study, adropin stimulated apoptosis in Gc. While adropin has shown anti-apoptotic effects in other tissues—e.g., stem cells [45]—its pro-apoptotic action in the ovary is supported by reports of increased Bax/Bcl-2 ratio and cleaved caspase-3 in mouse follicles [14]. Autophagy may also contribute to Gc fate regulation. Autophagy maintains cells survival and metabolic balance under stress by degrading and recycling cellular components. Key regulators include beclin1 (phagosome initiation), LC3 (autophagosome formation), and LAMP1 (autophagolysosome fusion) [46]. In our study, adropin reduced the expression of these markers, suggesting it may suppress autophagy in Gc, contributing to their degeneration. Briefly, adropin-induced downregulation of beclin1 and LAMP1, along with increased caspase 3 expression and its activation, suggests a shift from autophagy to apoptosis [47]. Similar roles have been reported for other adipokines: resistin modulates autophagy-related proteins in porcine luteal cells [48], and chemerin induces autophagy while promoting proliferation and inhibiting apoptosis in COV434 cells [49]. Together, these findings suggest that adipokines may coordinate proliferation, survival, and autophagy in a context-dependent manner. Nevertheless, the concomitant decrease in Gc viability and increase in cleaved caspase-3 expression suggest that the reduced proliferative activity cannot be attributed solely to differentiation-associated cell-cycle withdrawal, but may also reflect impaired cell survival.

In this study, we applied a dose-dependent approach, including a physiologically relevant adropin concentration (0.1 nM), close to its level in FF (~0.4 ng/mL). Higher doses (1 and 10 nM) were also used to explore broader biological effects and simulate pathophysiological conditions. For instance, adropin levels are elevated in hyperthyroidism, which impairs ovarian function [50], while in PCOS adropin level is reduced [4], suggesting possible therapeutic relevance of adropin supplementation. Our results indicate that adropin affects various cellular processes and gene/protein expression in a dose-dependent manner. The most pronounced effects were observed at doses 1 and 10 nM, while 0.1 nM was largely ineffective in most assays. For example, adropin reduced Gc viability only at 10 nM, but inhibited proliferation at all tested concentrations; these differences may result from varying sensitivities of specific signaling pathways, receptor activation thresholds, or differential receptor internalization and downstream signaling dynamics depending on the dose. It is also possible that different processes (e.g., proliferation vs. viability or steroidogenesis) require distinct levels or durations of receptor activation. A similar dose-dependent specificity has been observed for other adipokines. For instance, vaspin affected steroidogenesis at doses of 0.1 and 1 ng/mL [40], while its anti-apoptotic effect was already evident at a lower dose of 0.01 ng/mL [22].

Changes in kinase phosphorylation rapidly mediate cellular responses in ovarian follicles, influencing proliferation and endocrine function. MAP3/1 (ERK1/2) supports oocyte maturation [51], AKT promotes cell survival [52], PKA is involved in hormonal regulation [53]. We observed a time-dependent stimulatory effect of adropin on the phosphorylation of AKT, ERK1/2, and PKA in Gc. Previous studies link adropin to key signaling pathways: it increases MAP3/1 and AKT phosphorylation in rat mesenchymal stem cells [45] and insulin-induced AKT activation in mouse muscle [9]. However, in mouse liver, adropin suppressed PKA activity [54], indicating cell type-specific effects. This variability is not unique—e.g., vaspin stimulates STAT3 phosphorylation in porcine follicular cells [40] but not in luteal cells [55]. To further clarify the functional relevance we investigated the involvement of AKT and ERK1/2 in adropin-induced Gc viability and proliferation. Conversely, PKA inhibition reversed adropin’s suppression of E2 synthesis, indicating PKA involvement in its steroidogenic action. These findings partly align with previous: adropin promotes proliferation in 3T3-L1 and human adrenal carcinoma cells via ERK1/2 and AKT pathways [31, 35]. In tilapia hepatocytes, adropin also increases cAMP and activates PKA [56]. The decreased viability observed in the PD98059-only group may result from suppression of basal ERK1/2 signaling, which is involved in Gc survival and cellular homeostasis, which was previously presented in various cell lines [57]. Overall, adropin modulates Gc function through kinase-dependent pathways, with effects shaped by cell type and signaling context.

Oocyte maturation is crucial for embryo development and fertilization. While pigs are important for agriculture in vitro fertilization protocols remain inefficient due to unique features of porcine oocytes—high lipid content, oxidative stress sensitivity, complex metabolism—which hinder in vitro maturation and embryo development [58]. In the study, we selected our research model based on previous publications that explored the effects of various hormones on porcine oocyte maturation in vitro [59]. Notably, Marchal et al. [60] demonstrated that, although oocytes from prepubertal gilts are less competent and meiotically advanced compared to those from adults, they can still develop into blastocysts capable of reaching full-term development. For the first time, we demonstrated the presence of adropin and GPR19 in porcine oocytes and cumulus cells, confirmed by immunofluorescence. Furthermore, adropin protein expression was elevated in both oocytes and cumulus cells after in vitro maturation, while GPR19 expression increased only in cumulus cells. The literature provides evidence on the dynamic expression of adipokines during in vitro maturation, e.g., adiponectin receptor 2 transcription is reduced during bovine in vitro maturation in COCs [61]. Interestingly, the adipokine system not only reflects but also influences oocyte maturation. Adiponectin was shown to inhibit oocyte maturation [61], while vaspin expression upregulated during maturation and promoted this process [24]. Based on our findings, we hypothesize that the observed increases in adropin and GPR19 protein levels are associated with a stimulatory effect on oocyte maturation. As expected, we observed that adropin positively influenced nuclear maturation of oocytes and increased P4 levels in the culture medium after 44 h. P4 is produced by cumulus cells during in vitro maturation, and its addition has been shown to promote meiotic resumption in bovine oocytes [62]. When considered together with the increased proportion of matured oocytes, the enhanced P4 secretion may be consistent with functional differentiation of cumulus cells and an improved capacity to support oocyte nuclear maturation. This interpretation parallels the more differentiated steroidogenic phenotype proposed for isolated Gc, although direct markers of cumulus-cell differentiation were not evaluated in the present study. Adequate energy supply is essential for meiotic progression, redox balance and biosynthesis during oocyte growth. Cytoplasmic maturation—including metabolic and mitochondrial remodeling—is critical, as defects can impair development despite normal nuclear maturation [63]. Lipid droplets, rich in triglycerides, serve as the main energy reserves in oocytes [64]. We demonstrated that oocytes matured in the presence of adropin exhibited a reduced number of lipid droplets, suggesting enhanced lipolysis and improved oocyte quality. Additionally, we observed an upregulation of *ATGL* mRNA, which encodes a key enzyme in lipolysis, and a downregulation of *ADRP*, involved in lipogenesis [65]. These findings are consistent with the metabolic role of adropin previously described in other systems; e.g., adropin was shown to suppress hepatic expression of lipogenic genes in mice [1]. However, our data also revealed an inconsistency regarding adropin’s effect on E2 levels. While E2 is physiologically required to stimulate ovulation [63], we found that adropin reduced E2 secretion, yet still promoted oocyte maturation. This paradox may be explained by the phase-specific action of adropin in the ovarian cycle; in immature mice, adropin was shown to stimulate E2 secretion [14], while in adult animals, it inhibited E2 release [43]. Reduced Gc proliferation and steroidogenesis do not necessarily indicate impaired oocyte competence, as follicular maturation requires coordinated remodeling and differentiation of follicular somatic cells. Additionally, adropin stimulated apoptosis in Gc while simultaneously improving oocyte maturation, these effects were observed in different experimental models (mature vs. immature pigs). Importantly, increased apoptosis in cumulus cells is generally associated with reduced oocyte quality and developmental competence [66–68], suggesting that the pro-apoptotic effect observed in Gc may not occur in the same manner in cumulus cells. Importantly, human studies demonstrated that mural Gc and cumulus cells from the same follicle may exhibit distinct apoptotic responses and expression patterns, suggesting different physiological roles and sensitivity to external molecular signals, with cumulus cells being more closely involved in oocyte maturation and acquisition of developmental competence [69].

In summary, this study identifies adropin and its receptor GPR19 as novel regulators of porcine ovarian follicle function, suggesting a role for adropin as a metabolic modulator of fertility. Despite limitations such as the in vitro design and interspecies differences, targeting adropin signaling may offer new strategies for improving swine reproduction. Additionally, insights into adropin’s role in oocyte maturation could help optimize assisted reproductive technologies in both veterinary and biomedical fields.

## Supplementary Material

Supplementary_materials_ioag174_figure_1

## Data Availability

All data are incorporated into the article and its online supplementary material.
