# Supplementary material for: Adropin effects on granulosa cell function and oocyte maturation in pigs
Source: Biol Reprod. 2026 Aug 10;115(4):1184–99. doi: 10.1093/biolre/ioag174 (PMC13669399; doi:10.1093/biolre/ioag174)

Adropin/ACTB in follicle (fig. 1)

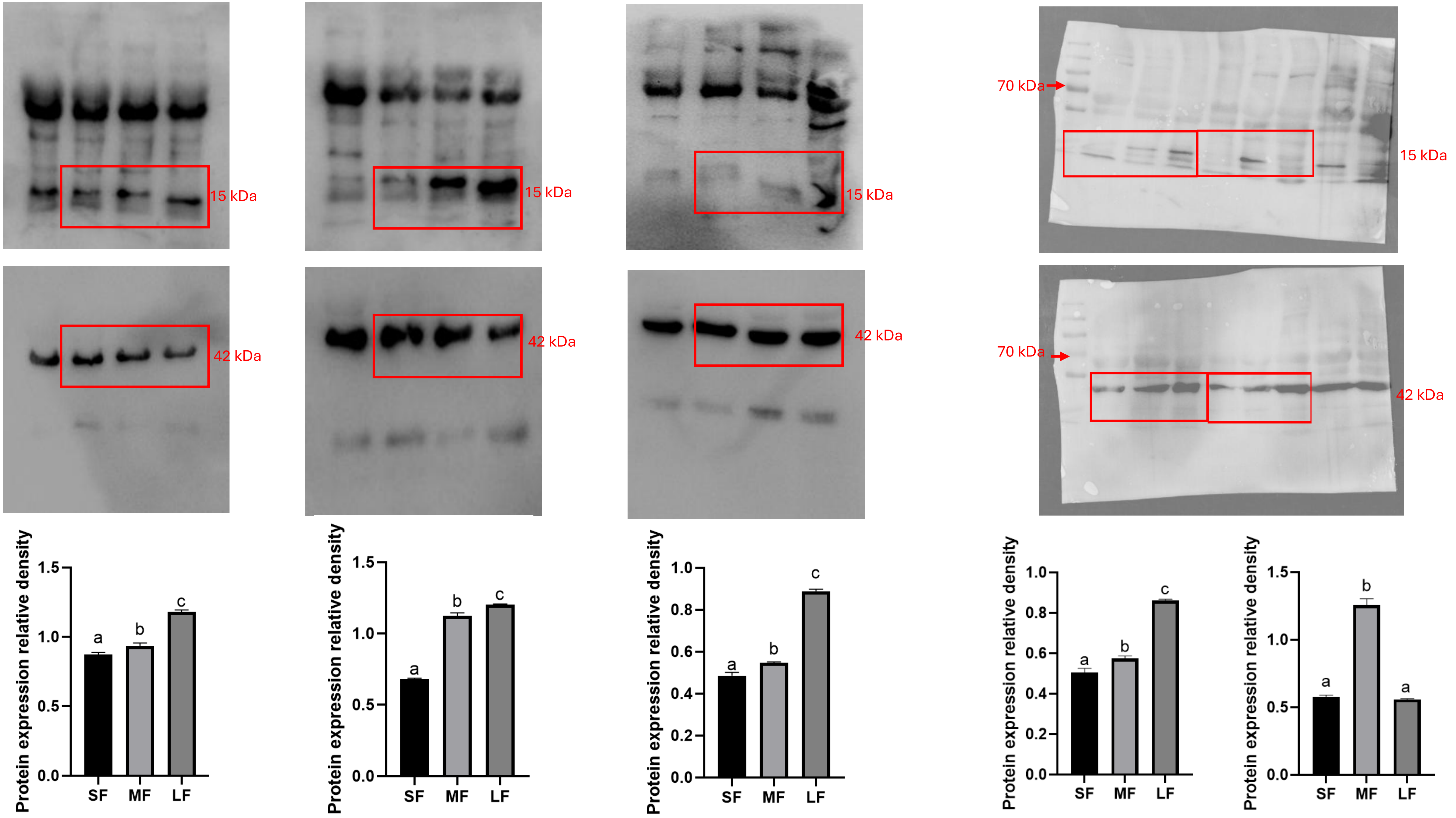

GRR19/ACTB in follicle (fig. 1)

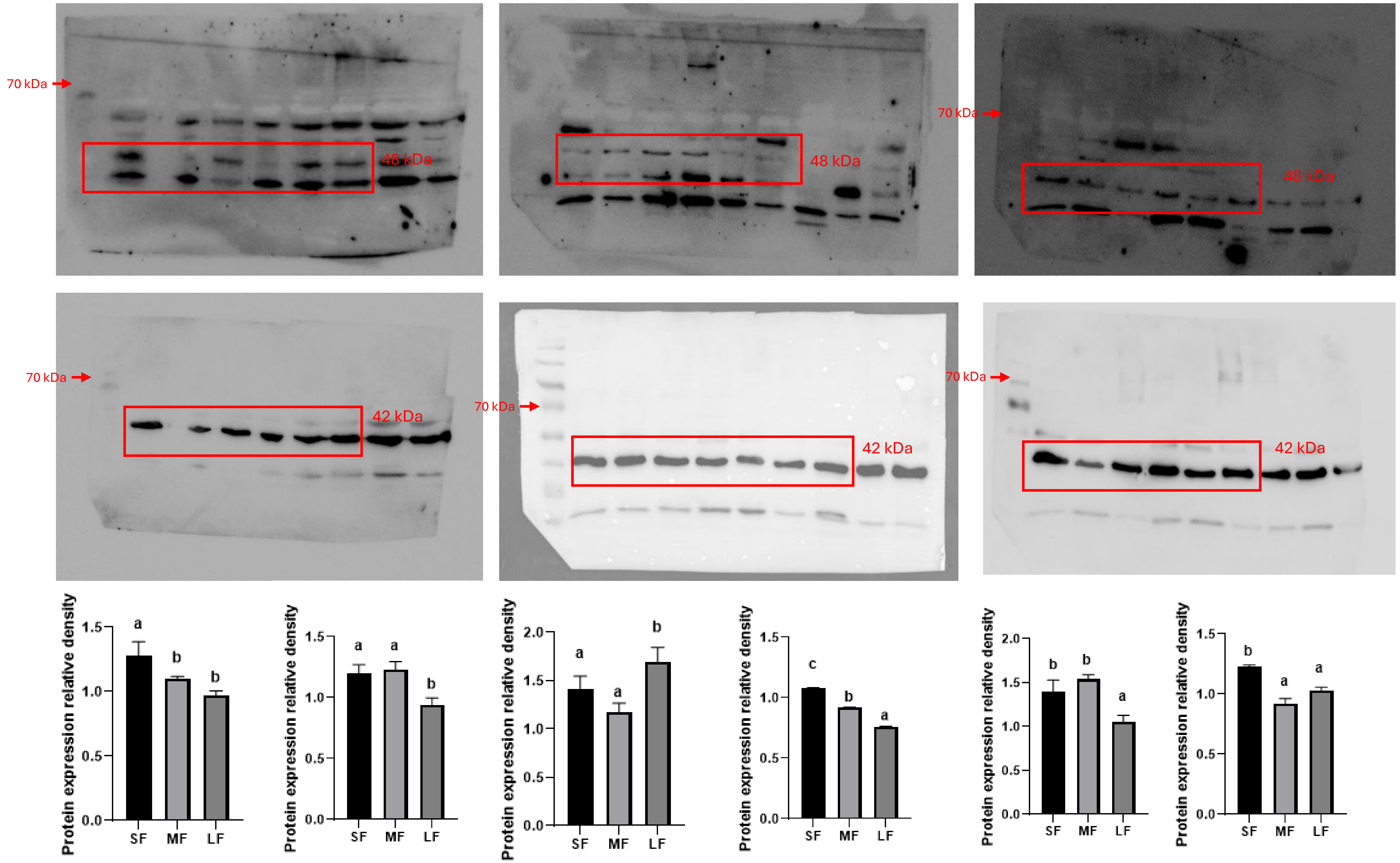

Adropin/ACTB expression regulation (fig. 1)

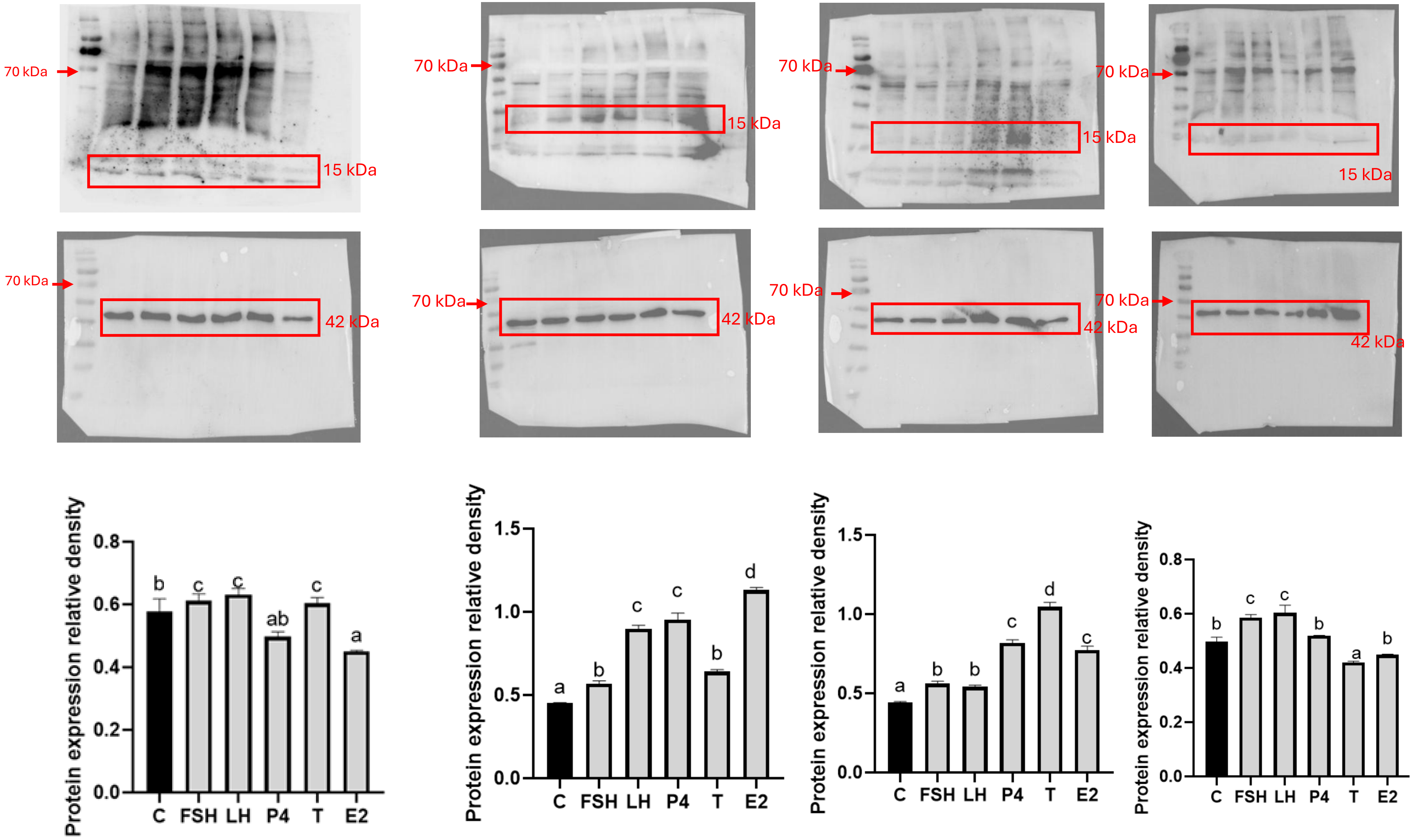

GPR19/ACTB expression regulation (fig. 1)

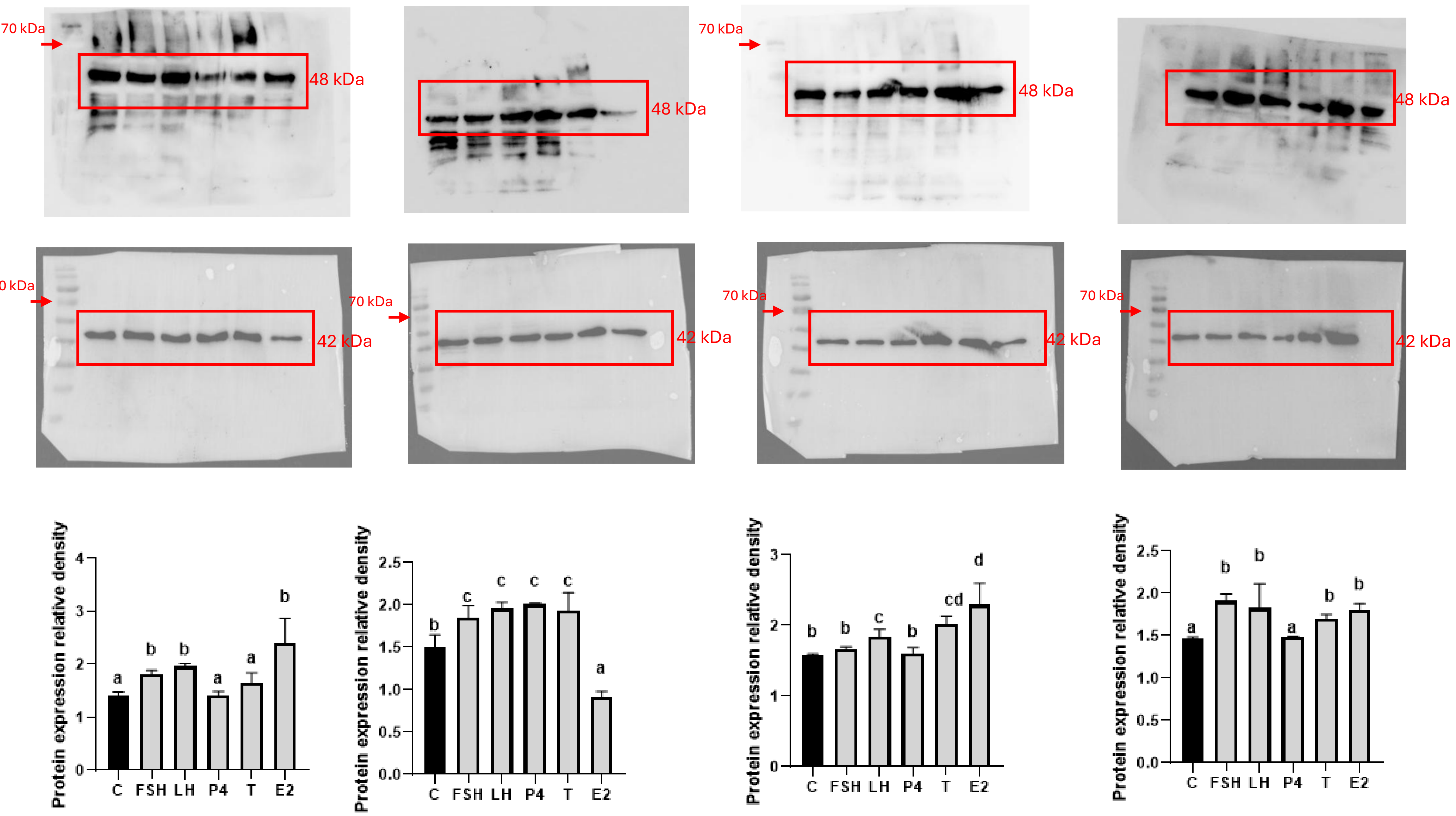

PCNA/ACTB (fig.2)

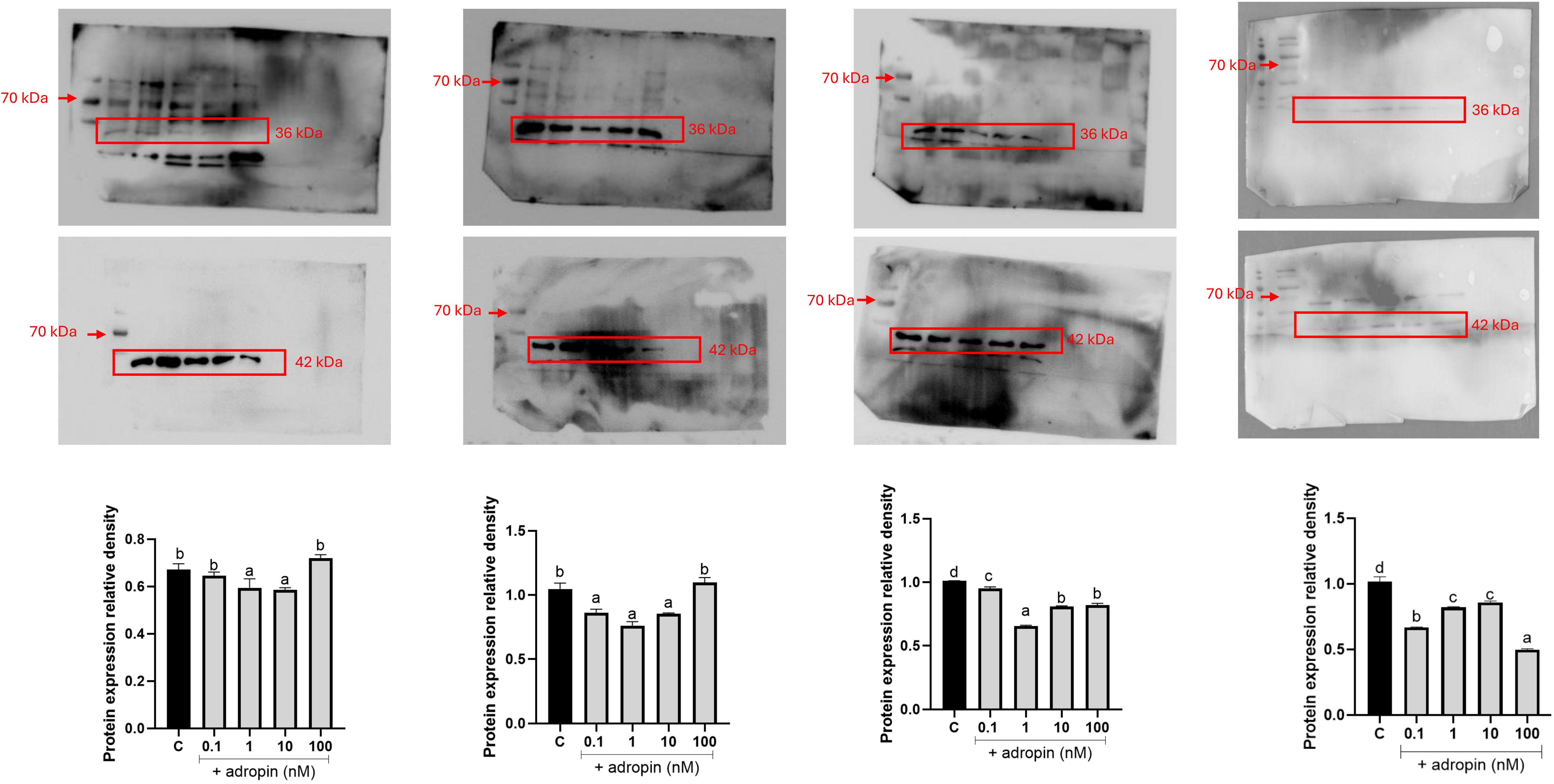

CyclinD/ ACTB (fig.2)

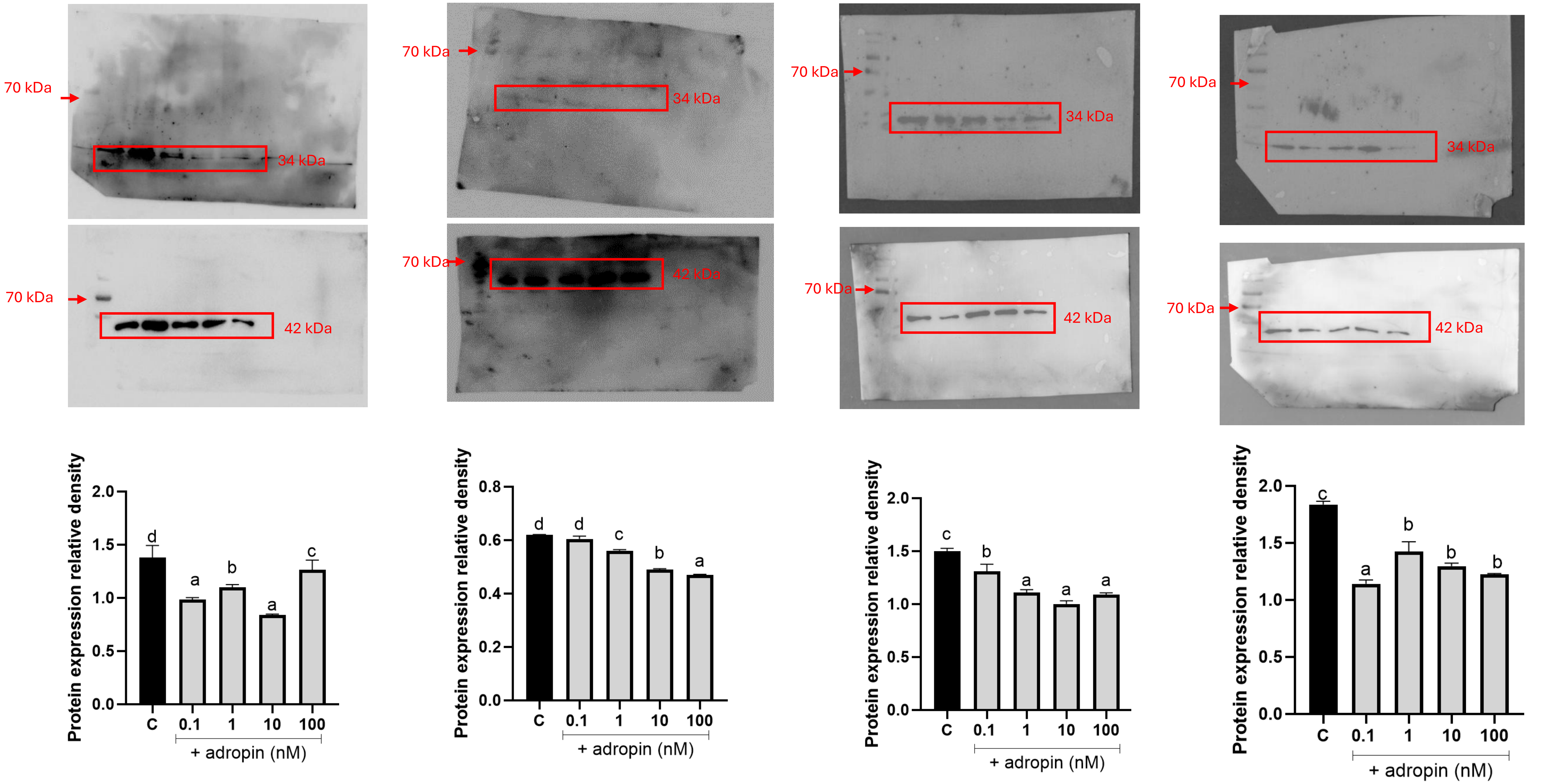

CYP19A1/ACTB (fig. 3)

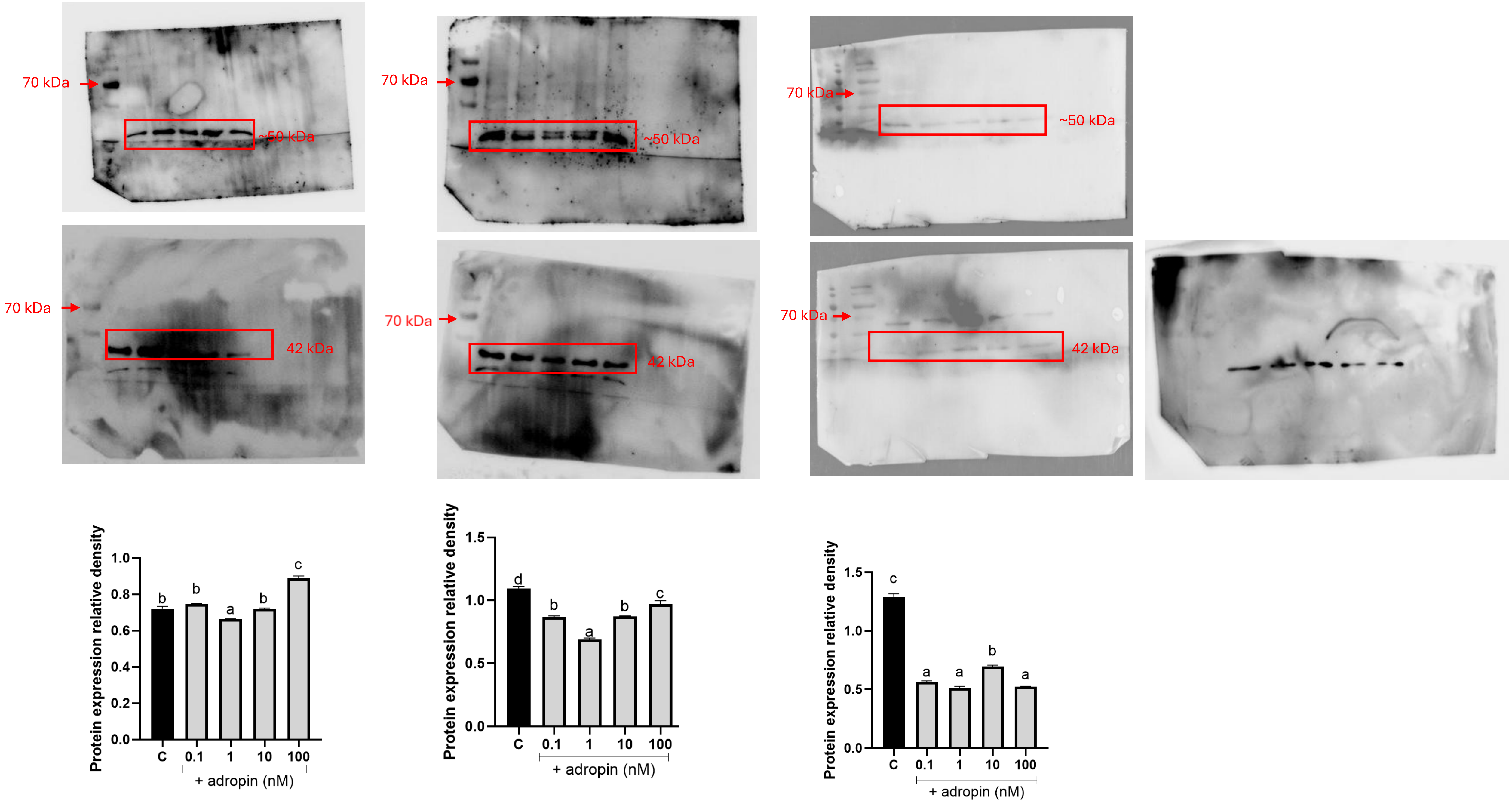

Caspase 3/ACTB (fig. 4)

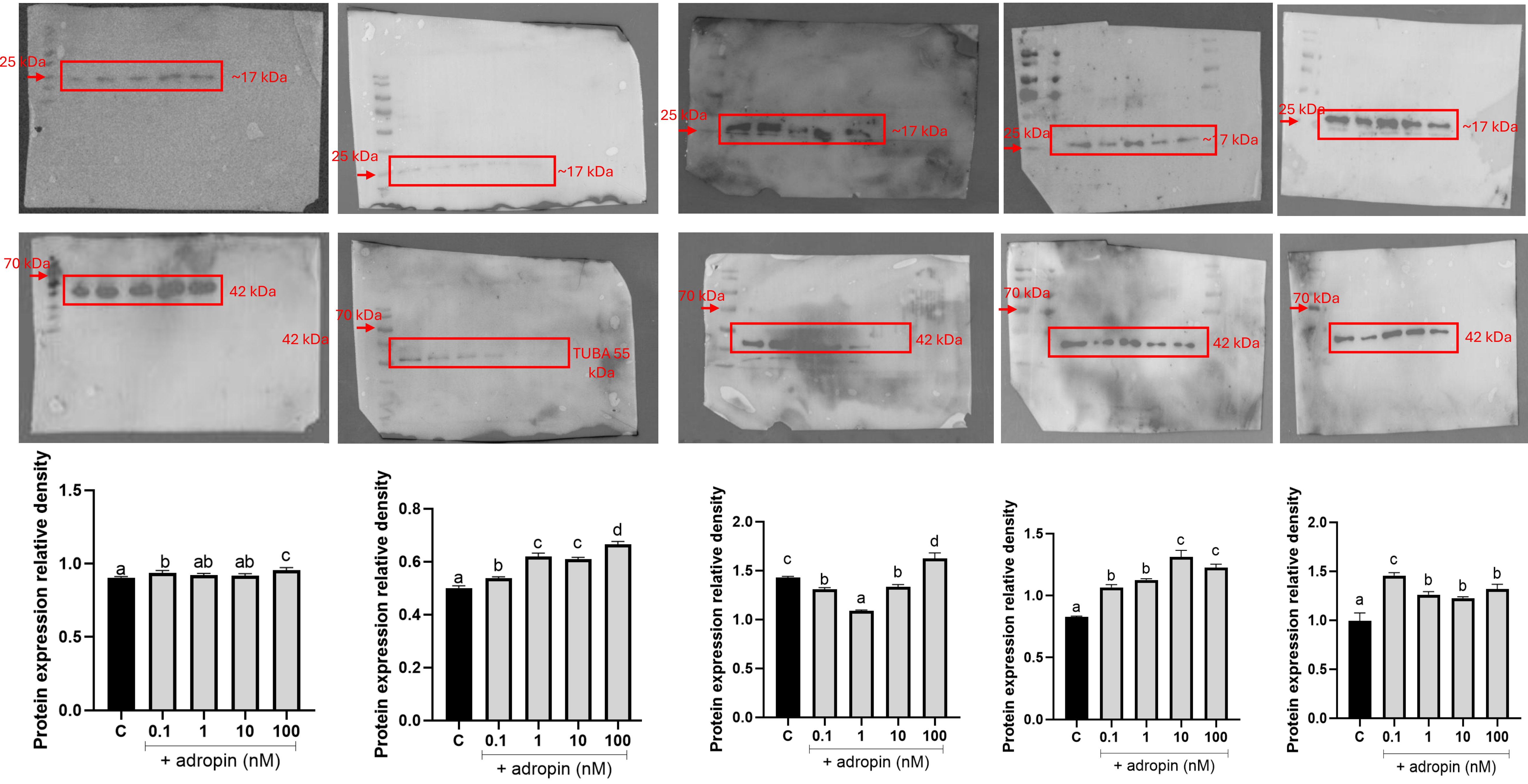

Beclin 1/ACTB (fig. 4)

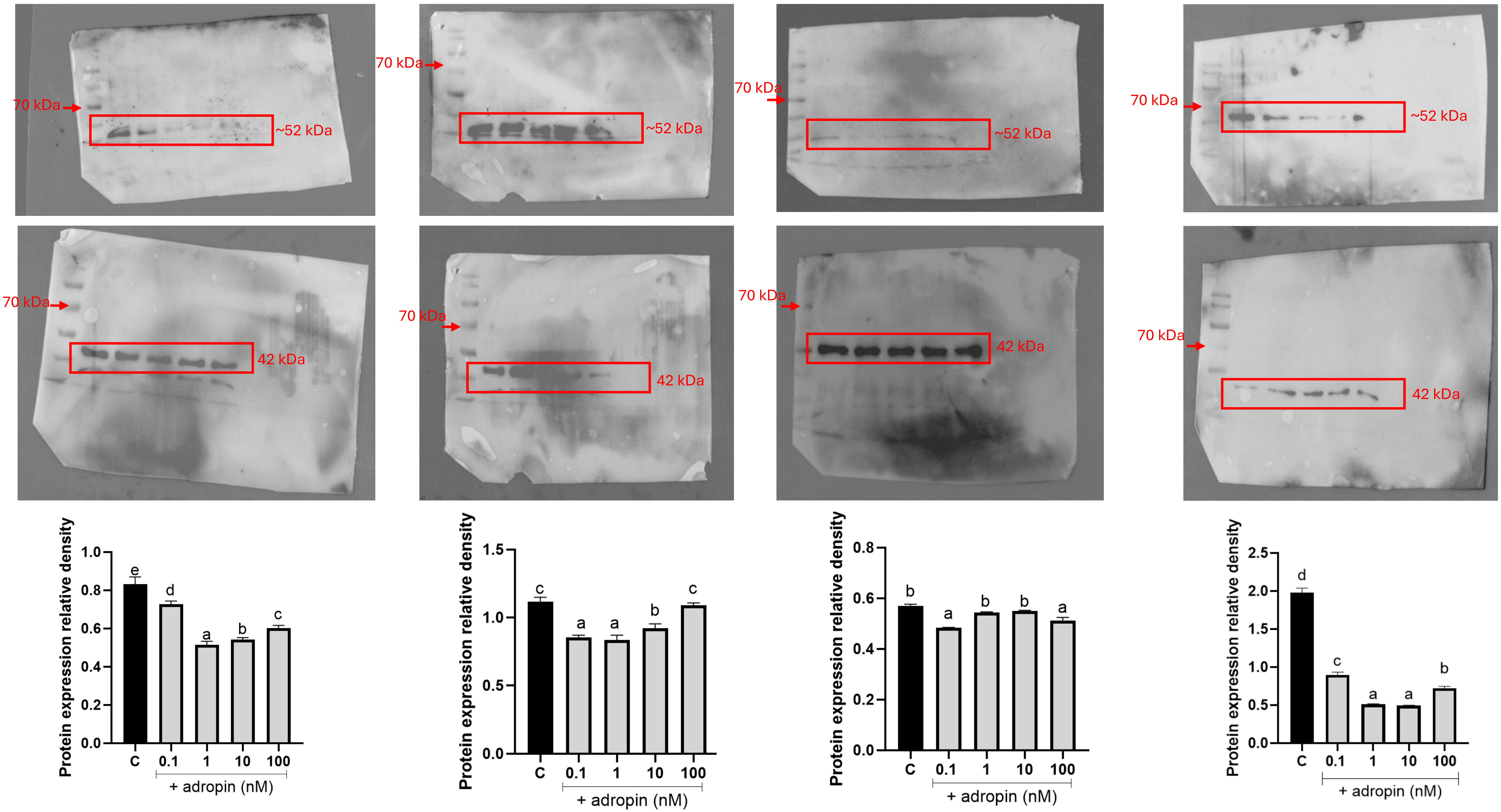

LC3/ACTB (fig. 4)

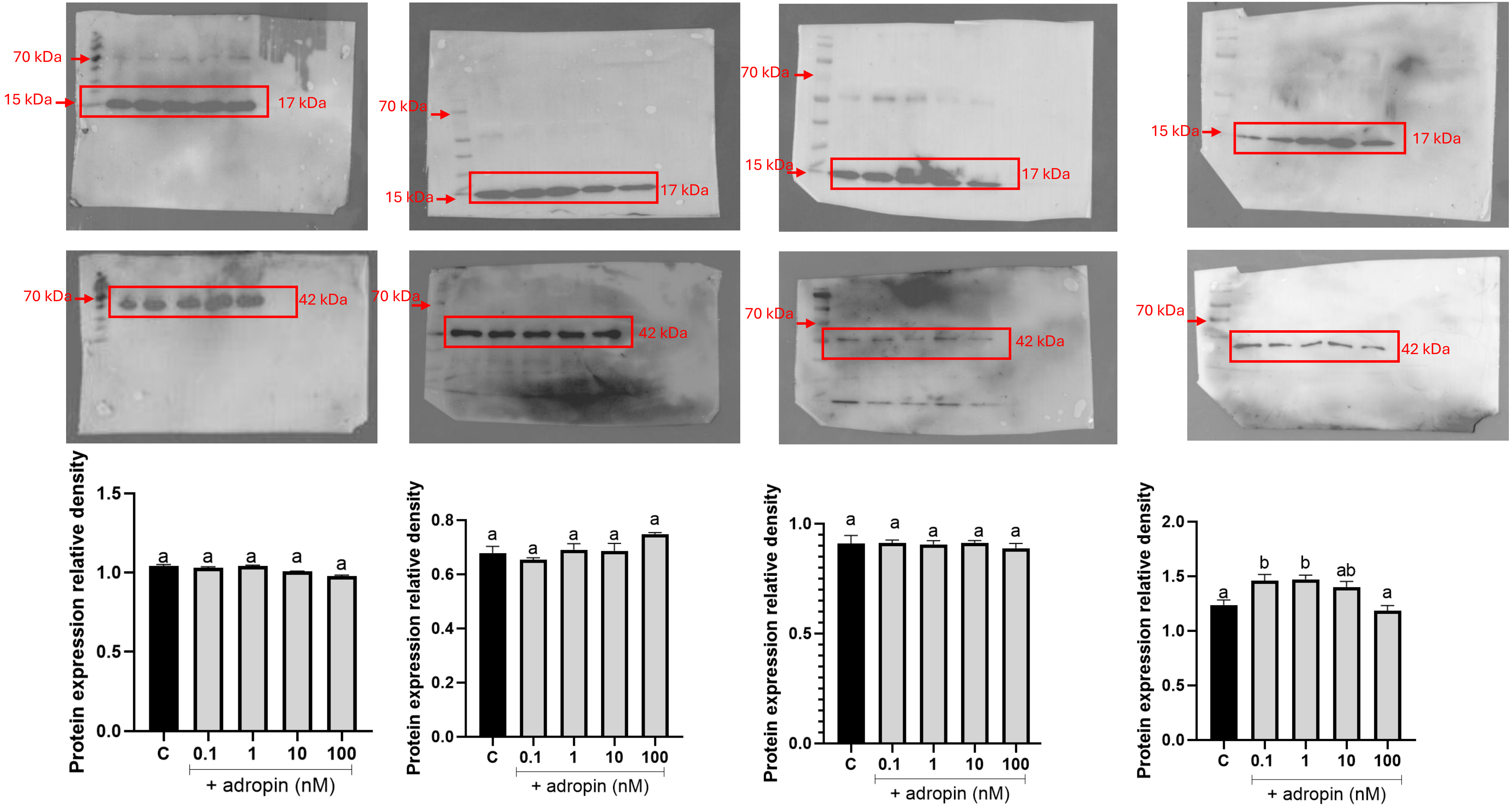

LAMP1/ACTB (fig. 4)

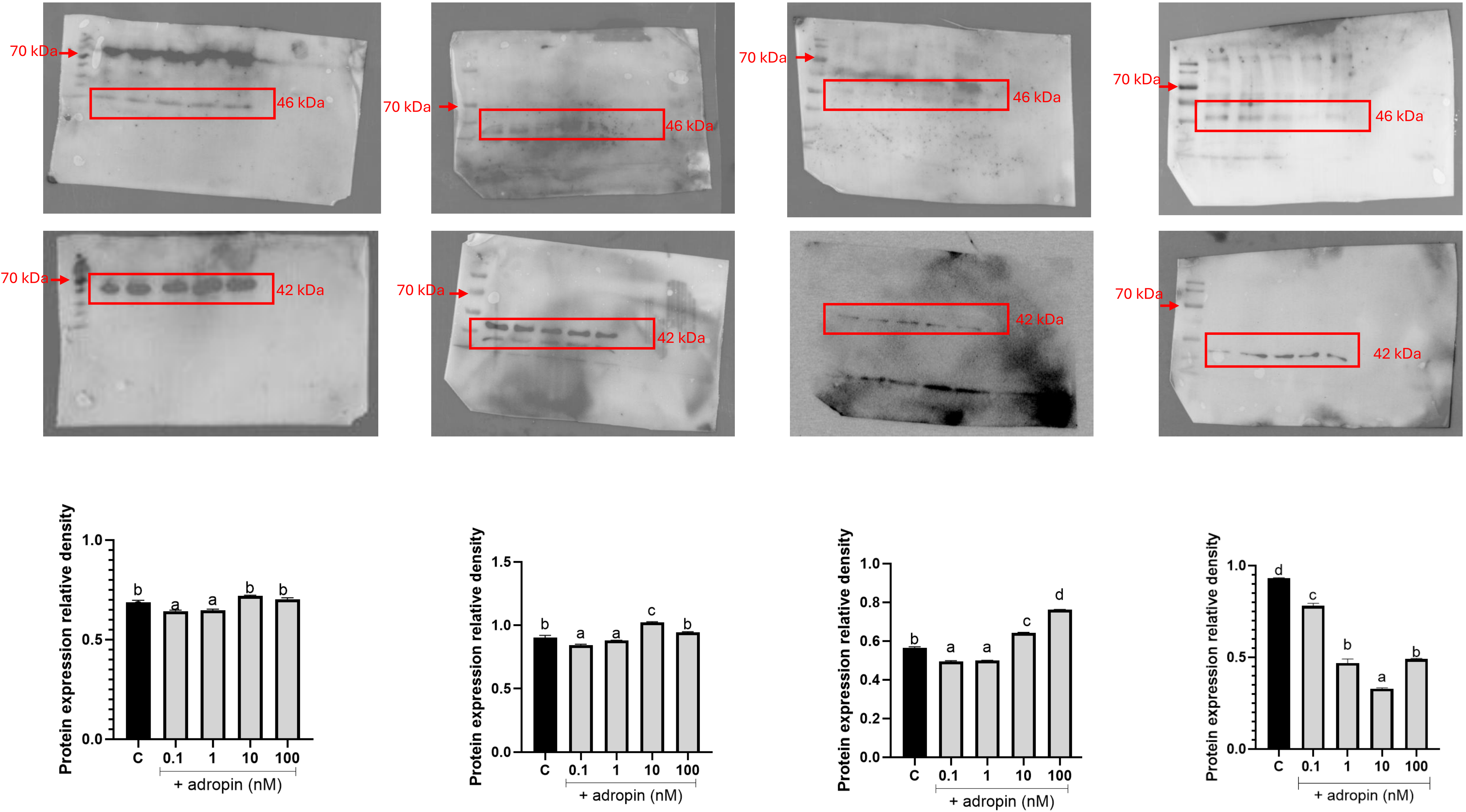

pAKT/tAKT (fig. 5)

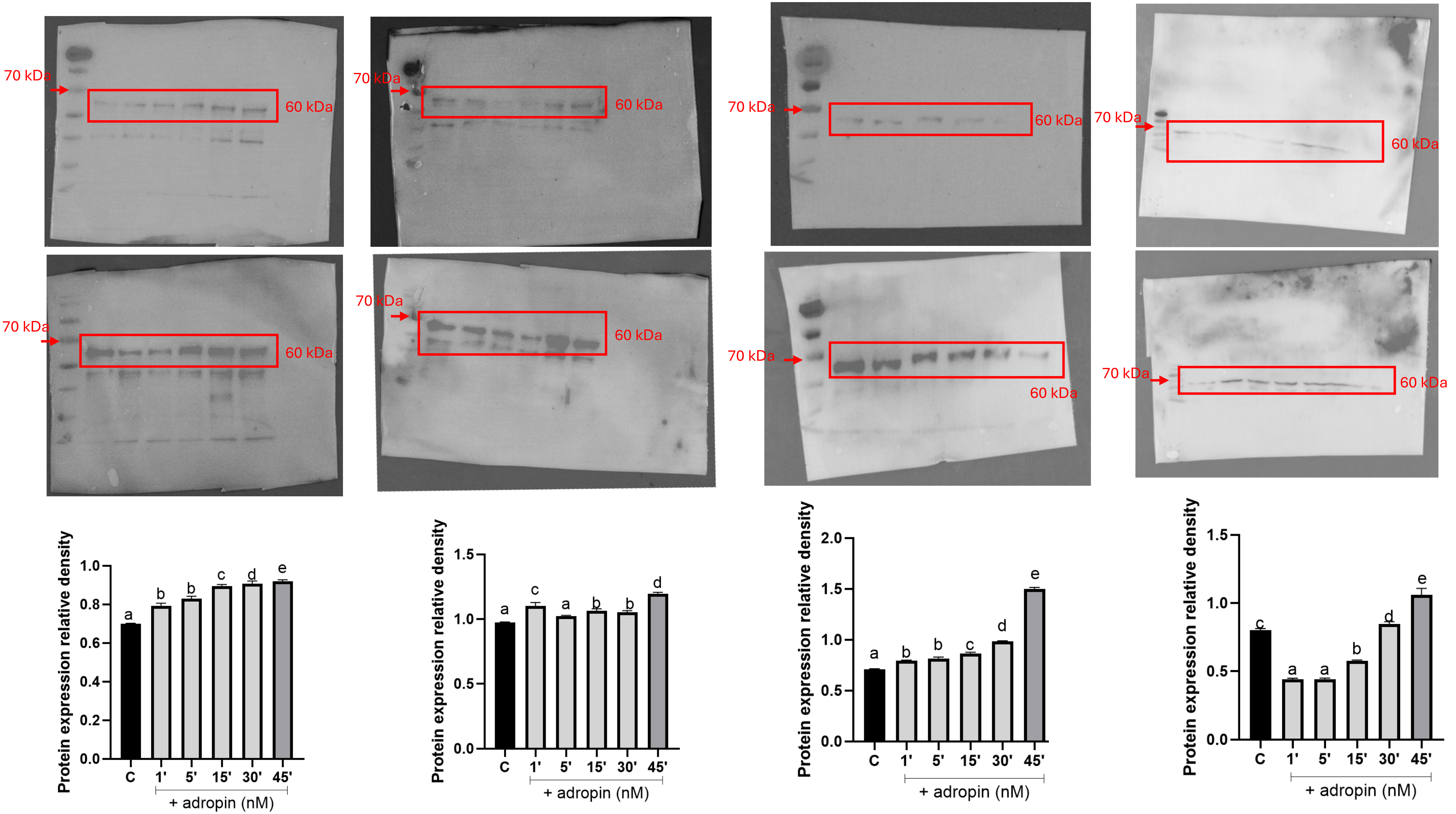

pERK1/2/ERK1/2 (fig. 5)

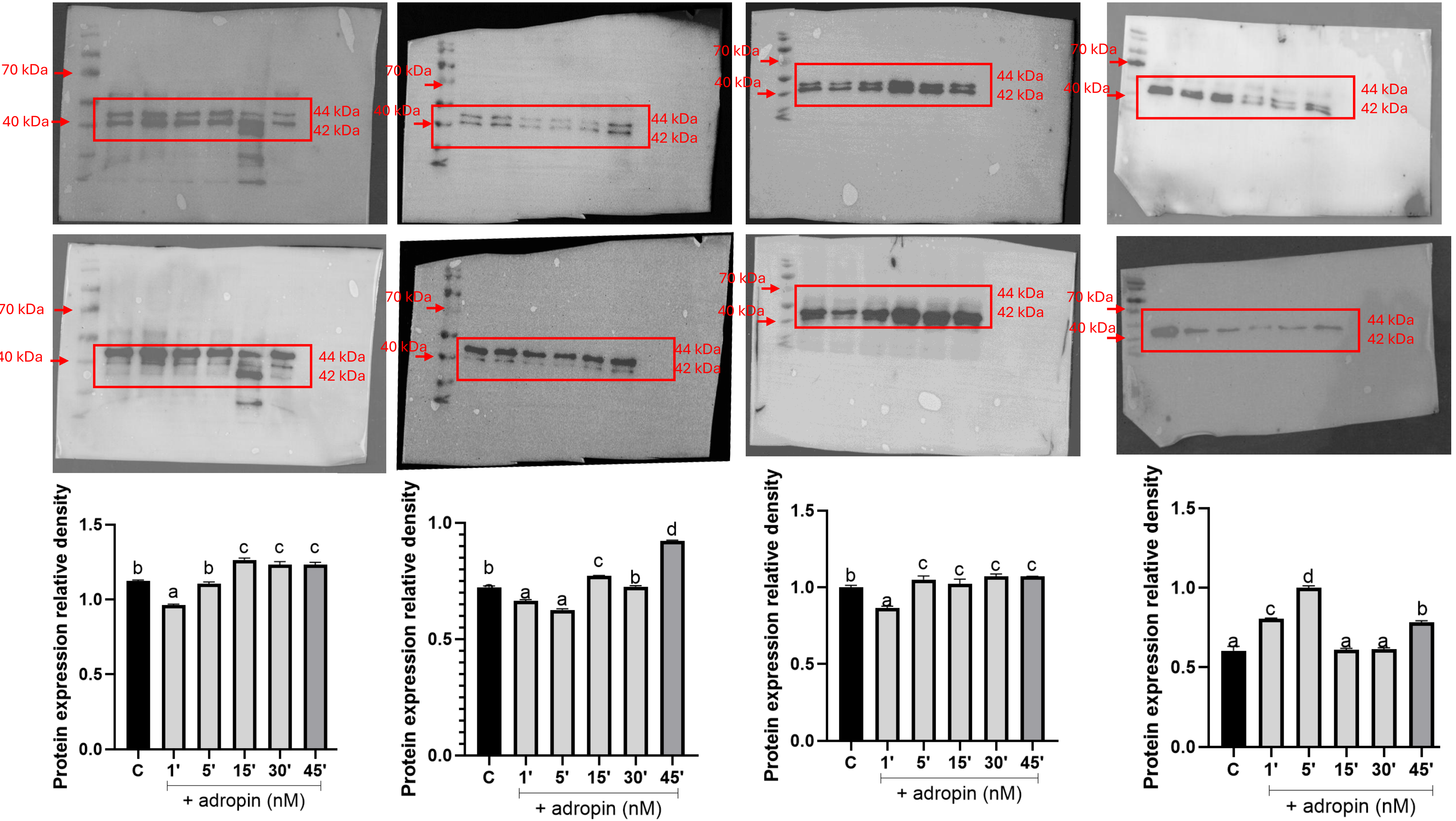

pPKA/ tPKA (fig. 5)

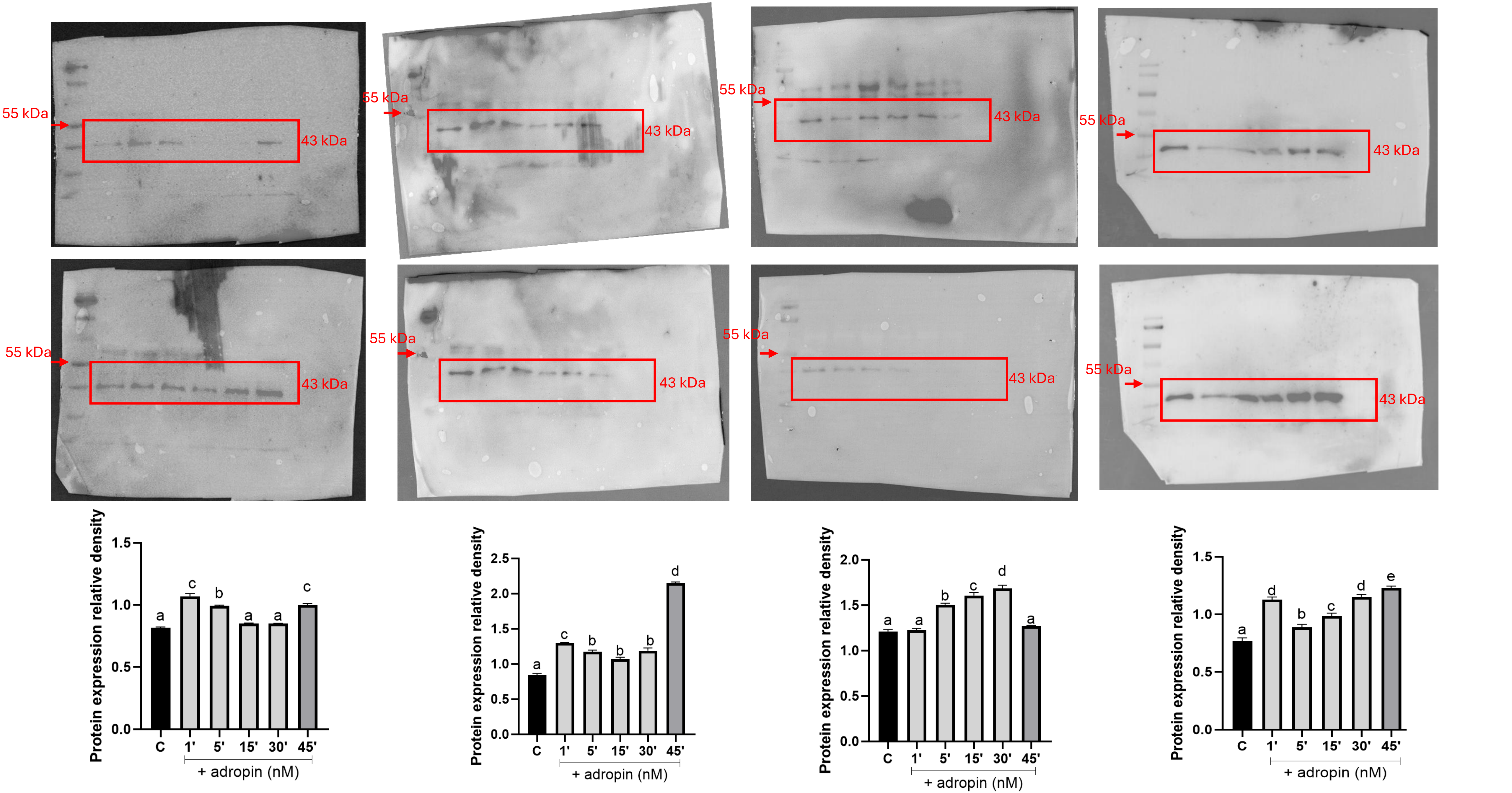

Adropin/ACTB in oocyte (fig. 6)

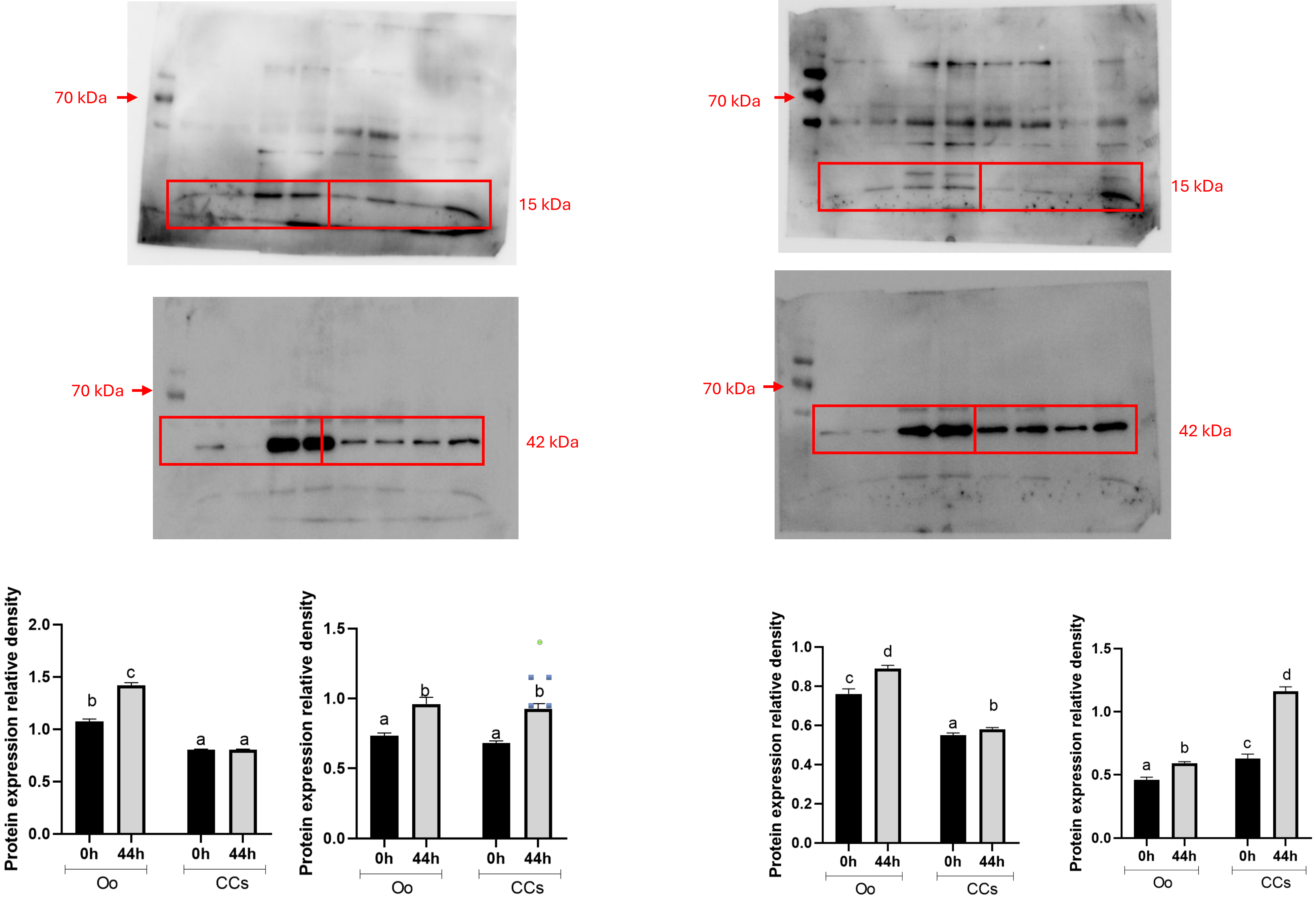

GPR19/ACTB in oocyte (fig. 6)

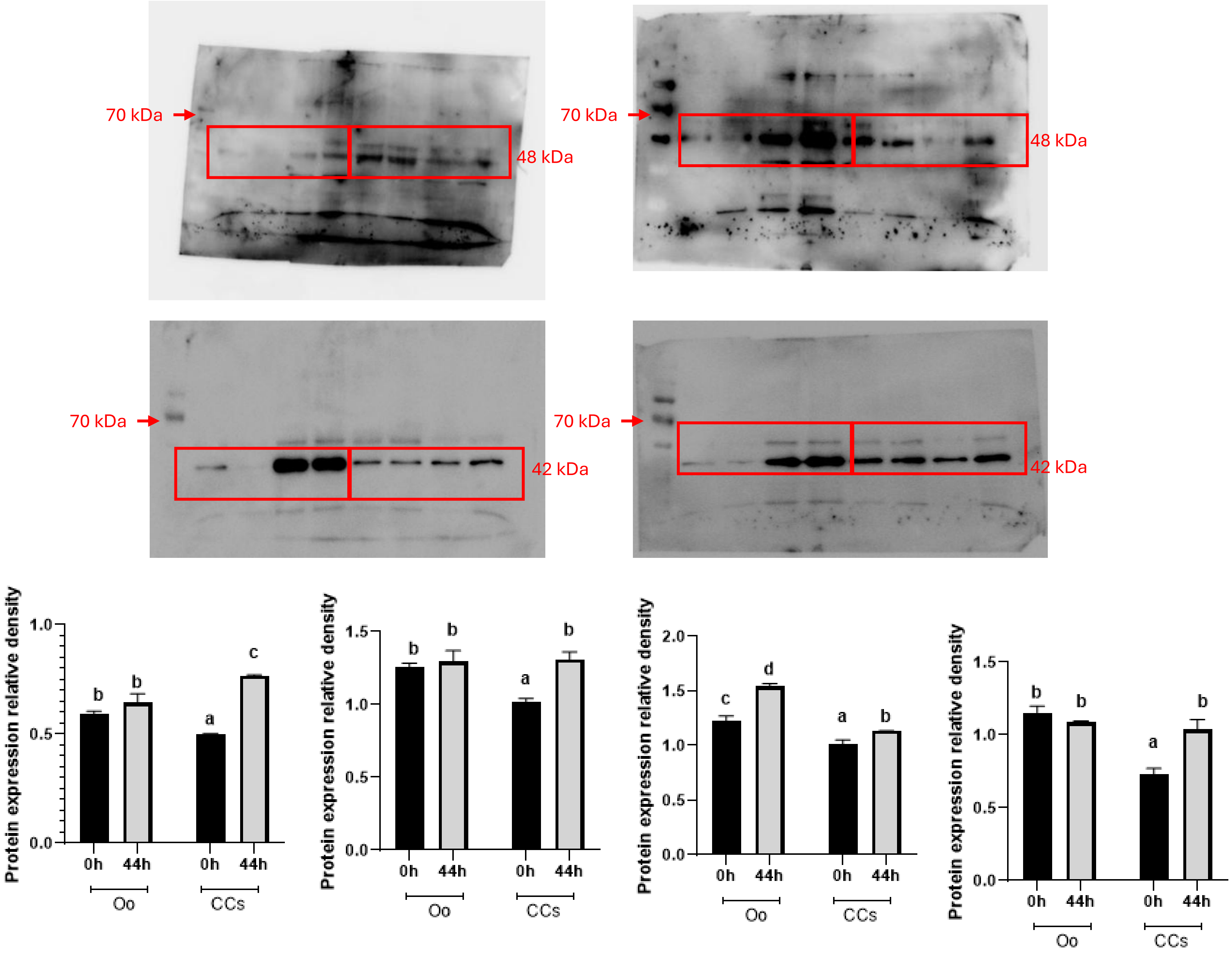

Supplement: Supplementary file 1 [file supplementary_materials_ioag174_figure_1.pdf]
